# Does aerobic exercise reduce NASH and liver fibrosis in patients with non-alcoholic fatty liver disease? A systematic literature review and meta-analysis

**DOI:** 10.3389/fendo.2022.1032164

**Published:** 2022-11-03

**Authors:** Veera Houttu, Julia Bouts, Yasaman Vali, Joost Daams, Aldo Grefhorst, Max Nieuwdorp, Adriaan G. Holleboom

**Affiliations:** ^1^ Department of Vascular Medicine, Amsterdam Gastroenterology, Endocrinology Metabolism, Amsterdam UMC, Location AMC at University of Amsterdam, Amsterdam, Netherlands; ^2^ Department of Experimental Vascular Medicine, Amsterdam Gastroenterology, Endocrinology Metabolism, Amsterdam UMC, Location AMC at University of Amsterdam, Amsterdam, Netherlands; ^3^ Department of Epidemiology and Data Science, Amsterdam UMC, Location AMC at University of Amsterdam, Amsterdam, Netherlands; ^4^ Medical Library, Amsterdam UMC, Location AMC at University of Amsterdam, Amsterdam, Netherlands

**Keywords:** non-alcoholic fatty liver disease, aerobic exercise, high-intensity interval training, moderate-intensity continuous training, systematic review, meta-analysis

## Abstract

**Background:**

Exercise is an effective strategy for the prevention and regression of hepatic steatosis in patients with non-alcoholic fatty liver disease (NAFLD), but it is unclear whether it can reduce advanced stages of NAFLD, i.e., steatohepatitis and liver fibrosis. Furthermore, it is not evident which modality of exercise is optimal to improve/attenuate NAFLD.

**Objectives:**

The aim is to systematically review evidence for the effect of aerobic exercise (AE) on NAFLD, in particular non-alcoholic steatohepatitis (NASH) and liver fibrosis.

**Methods:**

A systematic literature search was conducted in Medline and Embase. Studies were screened and included according to predefined criteria, data were extracted, and the quality was assessed by Cochrane risk of bias tools by two researchers independently according to the protocol registered in the PROSPERO database (CRD42021270059). Meta-analyses were performed using a bivariate random-effects model when there were at least three randomized intervention studies (RCTs) with similar intervention modalities and outcome.

**Results:**

The systematic review process resulted in an inclusion a total of 24 studies, 18 RCTs and six non-RCTs, encompassing 1014 patients with NAFLD diagnosed by histological or radiological findings. Studies were grouped based on the type of AE: moderate-intensity continuous training (MICT) and high-intensity interval training (HIIT). A total of twelve meta-analyses were conducted. Compared to controls, MICT resulted in a mean difference (MD) in the NAFLD biomarkers alanine transaminase (ALT) and aspartate aminotransferase (AST) of -3.59 (CI: -5.60, -1.59, p<0.001) and -4.05 (CI: -6.39, -1.71, p<0.001), respectively. HIIT resulted in a MD of -4.31 (95% CI: -9.03, 0.41, p=0.07) and 1.02 (95% CI: -6.91, 8.94, p=0.8) for ALT and AST, respectively. Moreover, both AE types compared to controls showed a significantly lower magnetic resonance spectroscopy (MRS) determined liver fat with a MD of -5.19 (95% CI: -7.33, -3.04, p<0.001) and -3.41 (95% CI: -4.74, -2.08, p<0.001), for MICT and HIIT respectively. MICT compared to controls resulted in a significantly higher cardiorespiratory fitness (MD: 4.43, 95% CI: 0.31, 8.55, p=0.03).

**Conclusion:**

Liver fat is decreased by AE with a concomitant decrease of liver enzymes. AE improved cardiorespiratory fitness. Further studies are needed to elucidate the impact of different types of AE on hepatic inflammation and fibrosis.

**Systematic Review Registration:**

https://www.crd.york.ac.uk/prospero/, identifier (CRD42021270059).

## 1 Introduction

The prevalence of obesity has strongly increased, driving an increase in the prevalence of non-alcoholic fatty liver disease (NAFLD) (1, 2). Consequently, NAFLD is now the most common liver disease globally, affecting 30–40% of adult men and 15–20% of adult women (3). The latest findings show an alarming number of children who are developing NAFLD in their early childhood (4). NAFLD is intertwined/associated with multiple metabolic diseases, i.e., metabolic syndrome and type 2 diabetes mellitus (T2DM). At least half of the patients with T2DM have NAFLD. Moreover, atherosclerotic cardiovascular disease (asCVD) is the main cause of mortality among patients with NAFLD (5–7).

The disease spectrum of NAFLD ranges from simple steatosis to non-alcoholic steatohepatitis (NASH) and fibrosis, of which the latter often results in liver-related mortality and morbidity (8, 9). NAFLD can even lead to cirrhosis and hepatocellular carcinoma (HCC), and patients with NAFLD might ultimately require liver transplantation (10–12). The pathophysiology of NAFLD is complex, but insulin resistance seems to be a crucial driving factor (13). Hyperalimentation (14), total parental nutrition (6), or sedentary lifestyle in combination with genetic heritability have also been recognized as important drivers of NAFLD (2, 15).

Despite the magnitude of the clinical problems of NAFLD and its burden on public health, pharmacotherapy for advanced stages of NAFLD has not yet been developed (16). Therefore, lifestyle interventions are still cornerstone management elements for NAFLD (17–19). Current guidelines targeting NAFLD recommend lifestyle therapies, including exercise and dietary modifications. Aerobic exercise (AE) is a type of physical activity when increase in the heart rate and breath are maintained over a period of time (20). Patients with hepatic steatosis are recommended to perform moderate-intensive or vigorous-intensive AE for 150–300 or 75–150 minutes/week, respectively (17–19). In addition to the direct liver related benefits of AE, AE reduces the risk for asCVD in patients with NAFLD (21). However, the evidence for the effect of AE on advanced stages of NAFLD, including fibrosis and NASH, is scarce. Furthermore, which type or modality of exercise intervention is optimal for patients with NAFLD is not yet evident (22, 23).

To address these knowledge gaps, we systematically reviewed the scientific literature to explore the effects of AE without dietary adjustments on NAFLD and NASH, and its associated markers. In this study, we focused on different modalities of AE, namely high-intensity interval training (HIIT), moderate-intensity continuous training (MICT) and sprint-interval training (SIT).

## 2 Methods and materials

This study was reported using the Preferred Reporting Items of Systematic Reviews and Meta-analysis (PRISMA) statement guidelines (24). The protocol of the systematic review is available in PROSPERO (CRD42021270059) (25).

### 2.1 Search strategy and data sources

A sensitive systematic literature search was conducted in Medline (*via* OVID) and Embase (*via* OVID) in close collaboration with an information specialist (JD) in February 2021. Also, a scoping search was conducted in SPORTDiscuss in response to the reviewer suggestion yielding four articles in population other than patients with NAFLD being irrelevant to this systematic review. The search was limited to articles published in English. The full search strategy is described in detail in **Supplementary Material 1**.

### 2.2 The eligibility criteria

#### 2.2.1 Inclusion and exclusion criteria

Studies were eligible if they fulfilled the following inclusion criteria (1): adults patients (≥ 18 years of age) with NAFLD/NASH (with or without fibrosis) diagnosed by histology (liver biopsy) or by non-invasive methods such as magnetic resonance imaging (MRI), ultrasonography (US) or vibration-controlled transient elastography (VCTE; FibroScan) (2); the main outcomes of interest were changes from baseline to the follow up on intrahepatic lipids (IHL), liver stiffness, fibrosis, steatohepatitis and/or inflammation (3); study intervention designed with at least one AE arm without dietary intervention. Studies that contained a dietary intervention combined with exercise were excluded. Studies were excluded if they were animal studies, case reports, case series, conference abstracts and letter/commentary studies. Studies were also excluded if they included subjects with excessive alcohol use, viral hepatitis or autoimmune hepatitis, Wilson’s disease or hemochromatosis, or when the subjects were children (< 18 years of age).

### 2.3 Screening process

After deduplication, the remaining titles and abstracts of the articles were screened independently by two reviewers (JB and VH) using the Rayyan QCRI program (26). In case of any disagreement, consensus was reached by discussion between the reviewers. In next screening phase, full texts were judged independently by the same two reviewers. A third reviewer (AH or YV) was consulted in case of disagreements.

### 2.4 Assessment of methodological quality

The Cochrane risk of bias tool for RCTs (RoB2) (27), and the Cochrane risk of bias tool (ROBINS-I) for non-RCT (28) were used to assess the risk of bias, which was performed by JB and VH independently. The risk of bias tool RoB2 contains five different domains that were used to assess the risk of bias, namely those 1) arising from the randomizing process; 2) due to deviations from the intended intervention; 3) due to missing outcome data; 4) in measurement outcome; and 5) in selection of the reported results. The overall judgment of the bias was classified based on the domains in RoB2 as a) low risk of bias, b) some concerns about bias, and c) high risk of bias.

ROBINS-I assessed 1) the risk of bias due to confounding effects; 2) the risk of bias in selection of participants into the study; 3) the risk of bias in classification of the intervention. The risk of bias tool ROBINS-I contains categories of low, moderate, serious and critical risk of bias, or no information.

### 2.5 Data extraction

The following study characteristics were extracted: title, author, country, year of publication, study design, diagnostic test features, study group characteristics, and the characteristics of exercise intervention (type of exercise, intensity and duration). Primary outcomes were NAFLD activity score (NAS) and individual histological scores for inflammation, ballooning and fibrosis, liver stiffness measurement (LSM) on FibroScan, liver fat (IHL based on MRI, score/grade based on US, steatosis based on controlled attenuation parameter (CAP) on FibroScan), and liver function markers (alanine transaminase (ALT), aspartate aminotransferase (AST), and gamma-glutamyl transferase (γGT)). Additional outcomes were glucose metabolism markers (glucose, insulin, hemoglobin A1c (HbA1c), and Homeostatic Model Assessment for Insulin Resistance (HOMA-IR)), plasma lipid profile markers (total cholesterol (TC), low-density lipoprotein cholesterol (LDL-C), high-density lipoprotein cholesterol (HDL-C), triglycerides (TG)), body composition (body weight, fat mass, body mass index (BMI), and (relative) amounts of visceral adipose tissue (VAT) and subcutaneous visceral tissue (SAT)) and cardiorespiratory fitness (peak or maximal oxygen uptake (VO2 peak) or (VO2 max)). Data extraction was conducted by one author and cross-checked by the other author (JB/VH). Study authors were contacted in case of the absence of reported values.

### 2.6 Statistical analysis

Included studies were categorized based on the type of the AE intervention (HIIT, MICT and SIT), the measurement technique of liver outcome (histology, MRI, US, FibroScan) and the type of control group. A meta-analysis was performed when there were at least three studies with similar intervention modalities and outcomes. In addition, outcome data were grouped based on intensity, type of AE (HIIT vs. MICT), and type of control group. When articles could not be included for meta-analysis, a narrative synthesis was used to summarize the findings.

Data per marker was unified by using appropriate conversion factors. In case HOMA-IR was not reported and data on glucose and insulin were available, HOMA-IR was calculated by using the formula HOMA-IR = *[fasting insulin x fasting glucose]/22.5*. Data expressed as mean with 95% confidence interval (CI) were calculated to standard deviations (SD) using the formula of the Cochrane Handbook for Systematic Reviews of Interventions (29). Changes after the intervention were calculated for each parameter in intervention and control groups according to the same Cochrane Handbook (29).

Meta-analyses were performed using the Cochrane Review Manager (RevMan version 5.4, the Cochrane Collaboration, 2020) (30). The extracted data were input as mean ± SD. Heterogeneity was checked by using the chi-square and I^2^ tests, and 95% CI was calculated using a random effects model. Pearson correlations of mean differences of two outcomes were performed by using IBM SPSS Statistics (version 26.0, Chicago, USA). Sensitivity analyses were conducted to investigate the influence of exercise duration, by removing a study with considerably longer intervention than other studies from meta-analysis (31).

### 2.7 Publication bias

Publication bias was reduced by searching in different electronic databases, checking abstracts for any further missing reports, checking references from other reviews and contacting experts and authors. Funnel plots were not constructed since the meta-analyses in this review do not have a required minimum of 10 studies per subgroup (29).

## 3 Results

### 3.1 Database search and article selection

**Figure 1:**
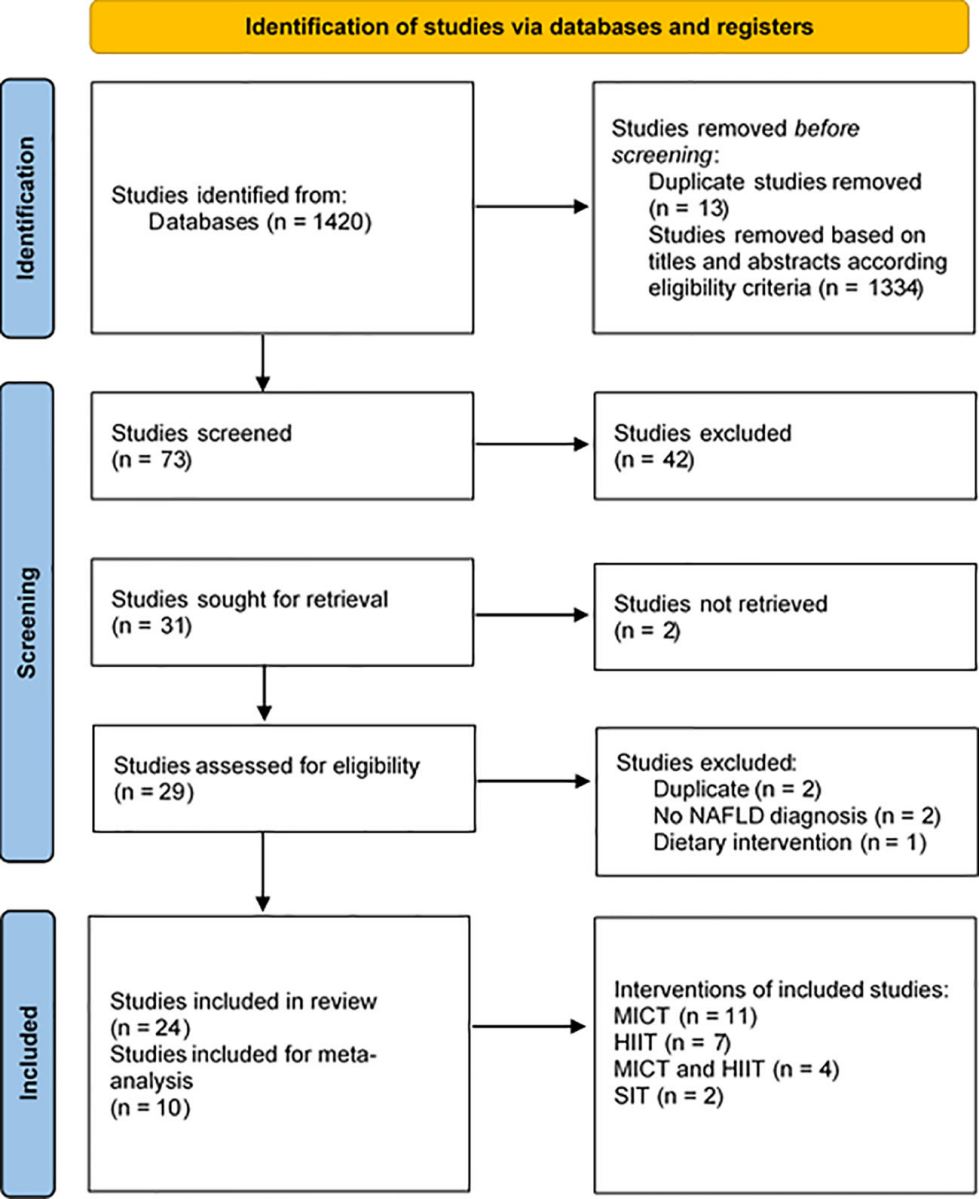
Preferred reporting items of systematic reviews and meta-analysis (PRISMA) flow chart [16]. HIIT, high-intensity interval training; MICT, moderate-intensity continuous training; SIT, sprint interval training.

The database search resulted in a total of 1420 articles. After screening the titles and abstracts, 73 remained for full-text assessment. According to the eligibility criteria, 24 studies, of which 18 were RCTs and six were non-RCTs, were included in this systematic review. In total, ten studies were included in the meta-analysis. The whole selection process is presented in the flow chart (**Figure 1**).

### 3.2 Study characteristics

This systematic review included 18 RCTs (**Table 1**) and six non-RCTs (**Table 2**), including a total of 815 and 199 subjects with diagnosed NAFLD, respectively. The sample size of the studies varied from 11 (43) to 209 (49). The mean age ranged between 39.7 ± 6.7 (39) and 60 ± 3.4 years (49), and most of the studies included both men and women. The method used for diagnosis of NAFLD and/or NASH varied: six studies used histology (n=121) (37, 41, 44, 46, 53, 55), nine MRI (n=399) (32, 33, 35, 43, 47–49, 51, 56), eight US (n=386) (34, 38, 42, 45, 46, 50, 52, 53), and one FibroScan (n=48) (39). Included studies reported NAFLD in different stages; histologically assessed NAS score varied from 3.6 (37) to 5 (41), while the patients’ steatotic status ranged from 10.3 ± 4.4% (40) to 31.3 ± 4.8% of liver fat (33) based on MRS. Liver stiffness was assessed by LSM FibroScan in three studies (n=68) (34, 42, 44), while two studies scored liver histology to assess hepatic fat and fibrosis (n=25), as well as liver inflammation and ballooning (37, 54).

**Table 1:** The characteristics of the included randomized controlled trials (RCT).

First author, year of publication and country	Intervention	Sample size (F/M)	Age (mean ± SD)	Disease stage	Diagnosis technique	Outcome assessment technique	Intervention details	Control	Duration
Abdelbasset et al., 2020, Saudi Arabia (32)	MICT and HIIT	I (HIIT): 16 (6/10) I (MICT): 15 (7/8) C: 16 (7/9)	I (HII): 54.4 ± 5.8 I (MIC): 54.9 ± 4.7 C: 55.2 ± 4.3	IHL % I (HII): 12.4 ± 4.5% I (MIC): 12.9 ± 4.2% C: 11.2 ± 5.1%	Diagnostic guidelines for NAFLD in the Asia-Pacific region	MRI-PDFF	3 x week, 40-50 min Cycling: 5-min warm up, continuous intensity at 60-70% of the max HR, 5-min cooling down 3 x week, 40 min Cycling: 5-min warm up, 4-min interval x 3 at 80-85% VO2max, 2-min rest intervals at 50% VO2max, 5-min cooling down	No exercise program, standard care	8 weeks
Bacchi, E. et al., 2013, Italy (33)	MICT	I: 14 (4/10) C: 17 (5/12)	I: 55.6 ± 2.0 C: 56.0 ± 1.9	IHL % I: 25.7 ± 3.7% C: 31.3 ± 4.8%	H-MRS	H-MRS	3 x week 60 min Treadmill, cycle or elliptical machines at 60-65% of heart rate	RT: 9 exercises on weight machines, 3 series of 10 repetitions at 70-80%	16 weeks
Cevik, T. et al., 2020, Turkey (34)	MICT	I: 16 (10/6) C: 15 (9/6)	I: 43.75 ± 8.62 C: 45.07 ± 9.11	–	US	FibroScan	4x week 40 min Cycle ergometer at 60-80% of HR	AE + whole body vibration: Vertical-sinusoidal vibration platforms, 15 min	8 weeks
Cheng, S. et al., 2017, China (35)	MICT	I: 22 (17/5) C: 18 (14/4)	I: 59 ± 4.4 C: 60 ± 3.4	NAFLD with impaired FG	H-MRS	H-MRS	2-3 x week, 30-60 min Nordic brisk walking + other group exercises at 60-75% of VO2max	No exercise program, Advised to maintain their current level of PA	12 weeks
Cuthbertson, D. et al., 2016, UK (36)	MICT	I: 30 (7/23) C: 20 (4/16)	I: 50 [46, 58]* C: 52 [46, 59]*	IHL % I: 19.4% [14.6, 36.1]* C: 16.0% [9.6, 32.5]*	Clinically by hepatologist	H-MRS	3-5 x week, 30-45 min Treadmill, cross-trainer, bike ergometer, rower at 30-60% of HRR	No exercise program, Advice about the health benefits of exercise in NAFLD	16 weeks
Eckard, C. et al., 2013, Italy (37)	MICT	I: 9 (3/6) C: 11 (4/7)	I: 51 ± 11 C: 52 ± 10	NAS I: 3.7 ± 1.1 C: 3.6 ± 1.1	Histology	Histology	4-7 x week, 20-60 min Exercise bicycle, treadmill	No exercise program, standard care	24 weeks
Franco, I. et al., 2019, Italy (38)	MICT	I: 42 (6/36) C: 52 (10/42)	Data not reported	Moderate to severe	US	US	4 x week, 45 min 65-75% of VO2	Combined exercise	24 weeks
Franco, I. et al., 2021, Italy (39)	MICT	I: 25 (11/14) C: 23 (6/17)	I: 50.45 ± 9.45 C: 46.23 ± 9.39	Moderate to severe	FibroScan	FibroScan	3 x week, 50-60 min Treadmill, cycling, cross-training and rowing at 60-75% MHR	Combined exercise	12 weeks
Hallsworth, K. et al., 2015 (40)	HIIT	I: 12 C: 11	I: 54 ± 10 C: 52 ± 12	IHL % I: 10.6 ± 4.9 C: 10.3 ± 4.4	Clinically	H-MRS	3 x week, 30-40 min Cycling: 5-min warm up (very light/somewhat hard), 2-min interval (very hard) x 5 + cumulative 10 sec increase per interval per week, 3-min rest, 3-min cooling down Intensity based on Borg rating of perceived exertion	No exercise program, standard care	12 weeks
Houghton, D. et al., 2017, Australia (41)	HIIT	I: 12 C: 12	I: 54 ± 12 C: 51 ± 16	NAS I: 5 [3, 7]* C: 5 [2, 7]*	Histology	H-MRS	3 x week, 45-60 min Cycling: 5-min warm up, 2-min interval x 3, 1-min rest intervals, Intensity based on Borg rating of perceived exertion (16-20, very hard) Resistant training: hip and knee extension, horizontal row, chest press, vertical row, and knee extension, Intensity based on Borg rating of perceived exertion (14-16, hard)	No exercise program, standard care	12 weeks
Oh, S. et al., 2017, Japan (42)	MICT and HIIT	I (HIIT): 20 (0/20) I (MICT): 13 (0/13) C (RT): 19 (0/19)	I (HIAT): 48.6 ± 1.8 I (MICT): 48.2 ± 2.3 C (RT): 51.2 ± 1.9	–	Diagnostic guidelines for NAFLD in the Asia-Pacific region	FibroScan CEUS	3 x week, 13 min Cycling: 2-min warm up (30 W, 60 rpm), 3-min interval x 3 at 80-85% VO2max (70-80 rpm), 2-min rest interval x 2 at 50% VO2max (60 rpm), 3-min cooling down (30 W, 60 rpm) 3 x week, 13 min Cycling: 2-min warm up (30 W, 60 rpm), 3-min interval x 3 at 80-85% VO2max (70-80 rpm), 2-min rest interval x 2 at 50% VO2max (60 rpm), 3-min cooling down (30 W, 60 rpm)	Resistance exercise: Push-ups, sit ups, leg press, leg extensions/curls, chest press, pull downs Intensity based on 1-RM strength test	12 weeks
Pugh, C. et al., 2013, UK (43)	MICT	Inclusion 13 (6/7) I: 6 C: 5	I: 50 [44, 56]* C: 48 [38, 57]*	–	US and elevated ALT, and H-MRS	H-MRS	3-5 x week, 30-45 min 30-60% HRR*	Conventional care	16 weeks
Rezende, R. et al., 2016, Brazil (44)	MICT	I: 19 (19/0) C: 21 (21/0)	I: 56 ± 7.8 C: 54.4 ± 8.9	–	Histology	FibroScan	2 x week, 30-50 min Treadmill	No exercise program	24 weeks
Shamsoddini, A. et al., 2015, Iran (45)	MICT	I (MICT): 10 (0/10) I (RT): 10 (0/10) C: 10 (0/10)	I: 39.7 ± 6.7 C: 45.8 ± 7.3	Moderate to severe	US	US	3 x week, 45 min Treadmill at 60-75% MHR (max HR)	No exercise program	8 weeks
Shojaee-Moradie, F. et al., 2016, UK (46)	MICT	I: 15 (0/15) C: 12 (0/12)	I: 52.4 ± 2.2 C: 52.8 ± 3.0	IHL % I: 19.6% [14.8, 30.0]* C: 12.5% [6.9, 32.9]*	US or histology	H-MRS	4-5 x week Gym-based aerobic exercise at 40-60%	Conventional lifestyle advice	16 weeks
Sullivan, S. et al., 2012, USA (47)	MICT	I: 12 (8/4) C: 6 (5/1)	I: 47.5 ± 2.2 C: 47.5 ± 3.1	IHL % I: raw data missing C:	H-MRS	H-MRS	5 x week, 30-60 min Treadmill at 45-55% VO2peak	No exercise program	16 weeks
Winn, N. et al., 2018, USA (48)	MICT and HIIT	I (HIIT): 8 I (MICT): 8 C: 5	I (HIIT): 41 ± 14 I (MICT): 46 ± 19 C: 51 ± 13	–	H-MRS	H-MRS	4 x week, duration was calculated as the ratio of 80 L/O2 by the average VO2 (L/min/O2) per each session Treadmill at 55% VO2peak 4 x week, Treadmill: 4-min intervals at 80% VO2peak, 3-min rest intervals at 50% VO2peak	No exercise program	4 weeks
Zhang, H. et al., 2016, China (49)	MICT and HIIT	I (MICT): baseline 73 (37/36) completed 69 (52/21) I (MICT-HIIT): baseline 73 (38/35) completed 66 (51/22) C: 74 (46/28)	I (MICT): 54.4 ± 7.4 I (MICT-HIIT): 53.2 ± 7.1 C: 54.9 ± 6.8	IHL % I (mod): 18.0 ± 9.9 I (mod-vig): 18.4 ± 9.9 C: 17.5 ± 11.0	H-MRS	H-MRS	5 x week, brisk walking 30 min (in total 150 min/week) at 45-55% MHR** Ttreadmill 30-min at 65-80% of MHR**	No exercise program	26 weeks

C, control group; CT, computed tomography; F, female; FG, fasting glucose; HIIT, high-intensity interval training; I, intervention group; M, male; MICT, moderate-intensity continuous training; MRS, magnetic resonance spectroscopy; Mri-PDFF, magnetic resonance imaging proton density fat fraction; NAFLD, non-alcoholic fatty liver disease; NASH, non-alcoholic steatohepatitis; PA, physical activity; RT, resistance training; SD, standard deviation; US, ultrasonography.

*95% CI.

**MHR, maximum predicted heart rate, calculated as 220/min for men and 210/min for women minus the participant’s age.

**Table 2:** The characteristics of the included non-randomized controlled trials (non-RCT).

First author, year of publication and country	Intervention	Sample size (F/M)	Age (mean ± SD)	Disease stage	Diagnosis technique	Outcome assessment technique	Intervention details	Control	Duration
Abd El-Kader, M. et al., 2014, Saudi Arabia (50)	MICT	50 (24/26)	I (AE): 50.87 ± 5.93 I (RT): 51.12 ± 5.58	–	US	NA	3 x week, 40 min 5 min warm-up, 30 min treadmill at 60%-80% of HRmax (increased weekly), 5 min cooling down	40 min, resistance machines, 10 min stretching, 30 min RT at 60 and 80% of their one maximal repetition weight	12 weeks
Haus, M.J. et al., 2013, USA (51)	HIIT	I: 14 (sex distribution not reported)	I: 55.6 ± 2.0 C: 56.0 ± 1.9	–	MRS	MRS	60 min for 7 days, treadmill at 85% of HRmax	–	7 days
Khaoshbaten, M. et al., 2013, Iran (52)	HIIT	I: 45 (16/29) C: 45 (17/28)	I: 35.6 ± 9.2 I: 39.5 ± 6.9	Grade I-III	US	US	30 min for 3 times x week, at HRmax	Medical therapy, 1000 mg vitC + 400 units vitE	3 months
MacLean, C. et al., 2018, UK (52)	SIT	1: 2 (2/10)	I: 45 ± 8	Steatosis to NASH	Histology or US	FIB-4	2 times x week (5–10 × 6-s ‘all-out’ cycle sprints)	NA	6 weeks
O’Gorman, P. et al., 2020, Ireland (53)	HIIT	I: 16 (12/4) C: 8 (5/3)	I: 61 ± 15 C: 58 ± 23	Steatosis to fibrotic NASH	Histology	Histology and FibroScan	2 supervised, 3 unsupervised times x week, 40-75% of HRR	Standard care	12 weeks
Sargeant, J. et al., 2018, Germany (54)	SIT	I: 9 (0/9)	I: 41 ± 8	IHL % 15.6 ± 8.4	H-MRS	H-MRS	3 x week, 4 intervals of max sprint cycling per session, increasing interval every 2 weeks (total of 90 intervals)	–	6 weeks

C, control group; CT, computed tomography; F, female; HIIT, high-intensity interval training; HRmax, maximum heart rate; HIIT, high-intensity interval training; HRR, heart rate reserve; I, intervention group; M, male; MICT, moderate-intensity continuous training; MRS, magnetic resonance spectroscopy; MRS-PDFF, magnetic resonance spectroscopy; NA, not applicable; NAFLD, non-alcoholic fatty liver disease; NASH, non-alcoholic steatohepatitis; NA, not available; RT, resistance training; SD, standard deviation; SIT, sprint interval training; US, ultrasonography.

### 3.3 Characteristics of exercise interventions

The AE interventions of RCTs and non-RCTs with varying types of sports, intensities and durations are presented in **Tables 1**, **2**. Of the 18 RCTs, four conducted HIIT, 10 MICT and four did both types of AE. There were three HIIT and one MICT non-RCTs. In two non-RCTs a sprint interval training intervention (SIT) was performed (53, 56). Altogether six studies executed a RCT-HIIT either by bicycle/ergometer with three training sessions per week with a duration per session varying from 13-min (42) to 40–60 min for 12 (40, 41) or eight weeks (32), or by treadmill training with four sessions per week for four weeks (48) or six months (49). Control groups received standard care or nothing, except for the resistance training (RT) control group of Oh et al. (42). Intensity of the training intervals was based either on VO2max (32), VO2peak (42, 48), Borg rating of perceived exertion (40, 41), maximum predicted heart rate (MHR) (49), heart rate reserve (HRR) (54) or maximum heart rate (HRmax) (51, 52).The duration of RCT-MICT interventions varied from eight weeks (32, 34, 45) up to six months (37, 38, 44, 49), and were conducted on a cycle ergometer (32, 34, 42) or a treadmill (33, 36, 37), or the intervention was treadmill or brisk walking (35, 44, 45, 47, 49). Interventions were performed at 60–80% (32–34, 38, 39, 42, 45) or between 30% and 60% of heart rate (HR) (36, 43, 46–49). Cardiorespiratory fitness was evaluated by ergospirometry or treadmill spirometry test in eight studies (32, 41–44, 46–48).

### 3.4 Meta-analysis and narrative review

The meta-analysis was performed for the liver outcomes (IHL, ALT, AST, γGT), glucose metabolism (glucose, HOMA-IR) and the plasma lipid profile parameters (TC, LDL-C, HDL-C, TG), as well as the cardiorespiratory fitness level (VO_2_max and VO_2_peak) and the total body weight from RCTs. Eight studies could not be integrated into the meta-analysis due to unsuitable study design or incomparable assessment techniques. Therefore, these eight studies are reported narratively. Among these studies is the one by Cevik et al. (34), in which two active arms, one AE intervention with and one without whole-body vibration, were used. Bacchi et al. (33) also performed a RCT with two active arms. Also, the 2019 and 2020 studies of Franco et al. (38, 39) had multiple active arms. The first study was conducted with two active arms and the latter with six active intervention arms. The studies by Oh et al. (42) and Winn et al. (48) had three and two active arms, respectively. For the studies of Eckard et al. (37) and Shamsoddini et al. (45) assessment techniques were not comparable with other studies. Additionally, all RCTs do not report/study of an outcome of interest, and therefore, are neither integrated in the meta-analysis nor reported narratively. All non-RCTs with the outcome of interest were narratively reviewed.

#### 3.4.1 Liver related outcomes

Various measures as a proxy of NAFLD/NASH have been used in the included studies. While most studies report plasma transaminases and use MRS to quantify IHL, some scored histology to assess inflammation and fibrosis, or performed FibroScan as a proxy for liver fibrosis.

##### 3.4.1.1 Inflammation and fibrosis

FibroScan using LSM was used only in two studies with a MICT intervention (n=35) (34, 44), thus meta-analysis of this method was not possible. In general, MICT did not result in a significant effect on LSM. In the HIIT study that employed LSM, the intervention led to a significant reduction in hepatic stiffness (-16.8%, n=20, p<0.005) (42). In another study, HIIT reduced LSM as well as histological assessed hepatocyte ballooning and fibrosis (54). A rather long 6-month MICT did not significantly reduce the histological-determined NAS (37).

##### 3.4.1.2 Liver transaminases

**Figure 2:**
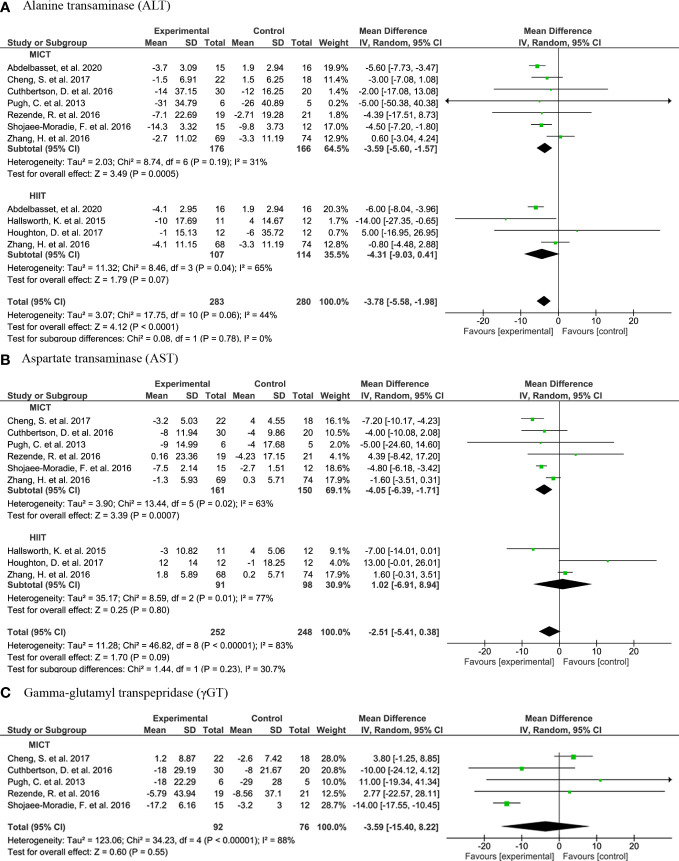
Forrest plot for the effect of moderate-intensity continuous training (MICT) and high-intensity interval training (HIIT) on liver transaminases: **(A)** alanine transaminase (ALT); **(B)** aspartate transaminase (AST); **(C)** Forrest plot for the effect of MICT on gamma-glutamyl transpeptidase (γGT).

With respect to liver transaminases, a meta-analysis of seven studies shows that subjects in AE (n=283) had significantly lower plasma ALT concentrations compared to controls (n=280) (MD: -3.78, 95% CI: -5.58, -1.98, p<0.001) (**Figure 2A**). However, the plasma ALT concentrations were only significantly lower in MICT subjects but not in HIIT subjects. Yet, there is no significant subgroup difference (I2 = 0%, Chi2 = 0.08, df=1, p=0.78). While plasma AST concentrations were not lower in AE compared to control, MICT subjects (n=161) had significant lower plasma AST concentrations than control subjects (n=150) (MD: -4.05, 95% CI: -6.39, -1.71, p=0.0007) (**Figure 2B**). No significant differences were observed between the groups in plasma γGT concentrations (**Figure 2C**).

Four RCTs that report transaminases were not included in the meta-analysis due to incomparable study designs. These studies showed varying results on the ALT concentrations upon AE. The study by Cevik et al. (34) reported a significant decrease in ALT and AST concentrations after an AE with and without whole body vibration from baseline. In turn, Bacchi et al. (33) did not show a significant reduction in ALT after an AE intervention (nor after RT intervention). In line, Winn et al. (48) did not find significant changes in transaminases from baseline after HIIT or MICT, but unfortunately, they did not report the change in ALT of the control group for comparison. Oh et al. (42) reported no change in ALT or AST concentrations after HIIT, but there was a significant change after MICT. In non-RCTs, there is a significant reduction of ALT and AST concentrations after the 3-month HIIT intervention in the study by Khaoshbaten et al. (52), as well as after a 12-week MICT intervention by Abd El Kader et al. (50). The SIT intervention by MacLean et al. (53) did affect transaminases. Houghton et al. (41) show close to significant reduction in γGT after 12-week HIIT. However, another 12-week HIIT did not results change in the concentrations (40), nor did AE with whole-body vibration (34).

##### 3.4.1.3 Intrahepatic lipids

**Figure 3:**
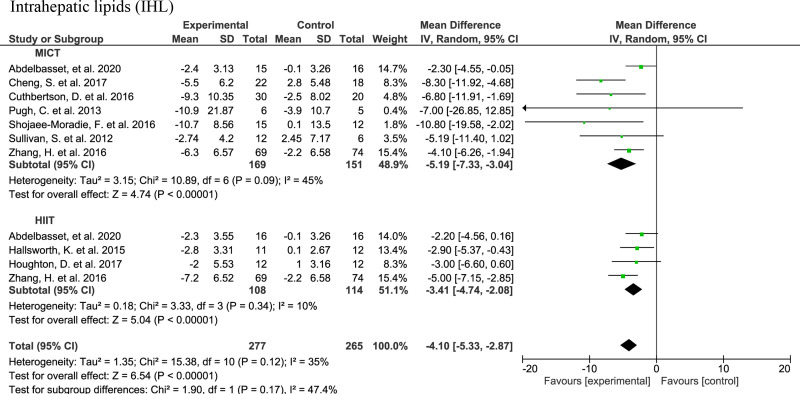
Forrest plot for effect of moderate-intensity continuous training (MICT) and high-intensity interval training (HIIT) on intrahepatic lipids (IHL) measured by magnetic resonance spectroscopy (MRS).

In total, eight studies were included in the IHL meta-analysis (**Figure 3**): five MICT studies (35, 36, 43, 46, 47), two HIIT studies (40, 41), and two studies with both HIIT and MICT (32, 49). There was a significant lower IHL upon the HIIT intervention (n=108) compared to the control (n=114) (-3.41; 95% CI: -4.74, -2.08, p<0.001). The IHL was also lower in the MICT subjects (n=169) than in the controls (n=151) (-5.19; 95% CI: -7.33, -3.04, p<0.001). The overall effect of AE (n=277), irrespective of the type, was significantly lower IHL than in controls (n=265) with a mean difference of -4.10 (95% CI: -5.33, -2.87, p<0.001).

Among the three RCTs that were not included in the meta-analysis due to large differences in the study design/intervention arms, Bacchi et al. (33) reported a 32.8% reduction of MRI-determined hepatic fat in AE arm compared to the baseline; another intervention arm was a resistance training showing a relative reduction of -25.9%. Moreover, Oh et al. (42) reported a reduction of hepatic fat after both HIIT and RT interventions of -16.6% and -47.2%, respectively. Also, Winn et al. (48) reported reductions in IHL of -37.0% or -20.0% after both HIIT or MICT, respectively. In non-RCTs, the SIT intervention by Sergeant et al. (56) led to 12.4% reduction of hepatic fat. O’Gorman and colleagues (54) do not report regression in steatosis assessed by histology after a 12-week HIIT. However, they observed a significant decrease in CAP measured by FibroScan (51). FibroScan was also used to assess liver steatosis measured using the CAP in three MICT studies (34, 42, 44). CAP was not significantly affected after an 8-week MICT intervention without whole-body vibration (34). In contrast, MICT with whole-body vibration showed significant decrease in CAP. The study by Oh et al. (42) showed a significant reduction in steatosis assessed using CAP after baseline in all three intervention groups (RT, HIIT and MICT) in accordance with MRI-determined IHL results.

#### 3.4.2 Body weight

**Figure 4:**
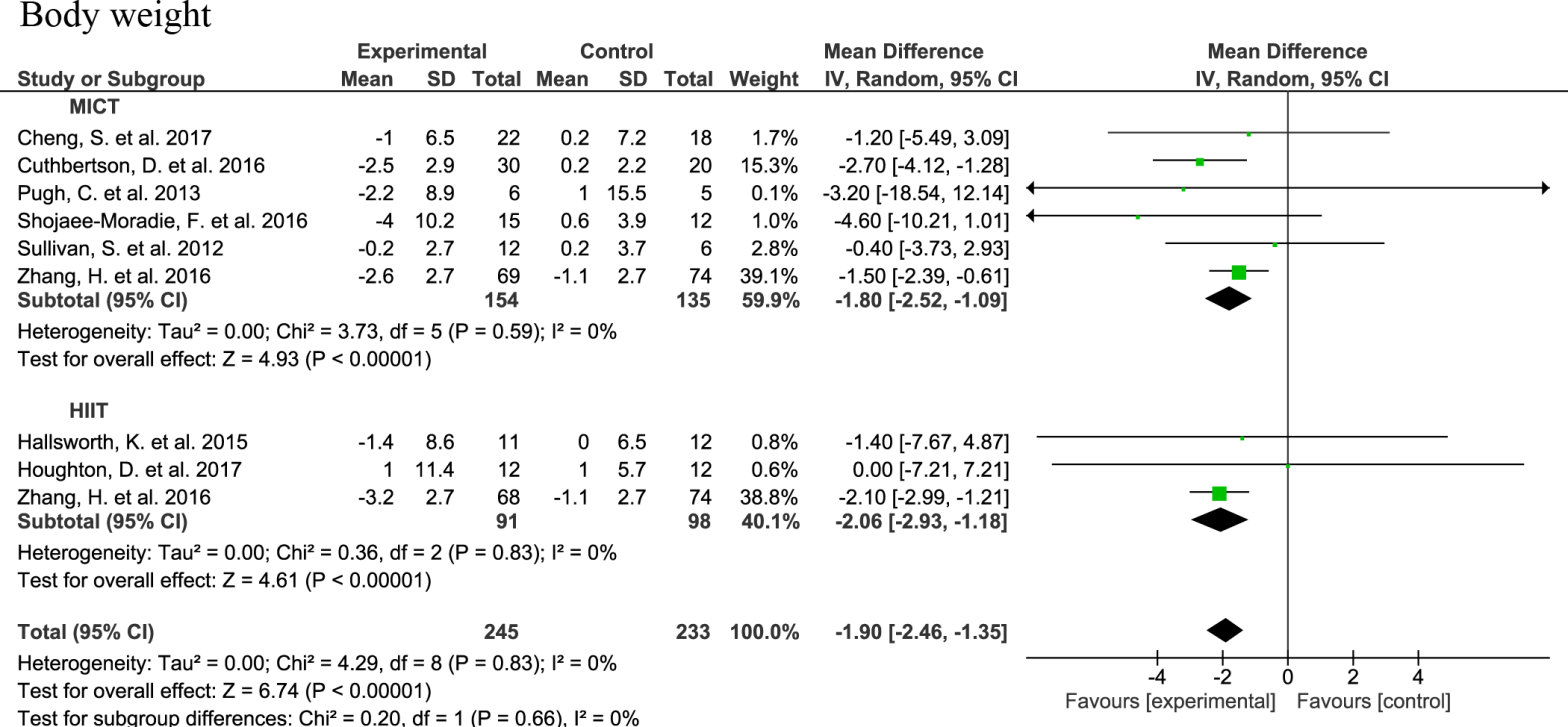
Forrest plot for the effect of moderate-interval continuous training (MICT) and high-intensity interval training (HIIT) on total body weight.

The body weight was lower upon AE compared to control with a MD of -1.90 (95% CI: -2.45, -1.35, n=245, p<0.001) without heterogeneity (I2 = 0, τ2 = 0.00, p=0.83). With respect to the AE subgroups, the MDs for body weight were -1.80 (95% CI: -2.15, -1.08, n=154, p<0.001) and -2.06 (95% CI: -2.93, -1.18, n=91, p<0.001) for MICT and HIIT, respectively. No significant subgroup difference was observed (Chi2 = 0.20, df=1, I2 = 0%, p=0.65) (**Figure 4**).

Studies that were not part of the meta-analysis used varying metrics to measure body compositional changes or did not have a comparable study design to be included in the meta-analysis. Among these the study of Abdelbasset et al. (32) reported a significant decrease in BMI after both HIIT and MICT. A MICT intervention by Rezende et al. (44) did not lead to a significant change in BMI, but it did decrease waist circumference, albeit that this was not significantly different from the decrease in the control group. Bacchi et al. (33) observed a significant decrease in BMI, total body fat mass, MRI-determined VAT and SAT, thickness of superficial subcutaneous adipose tissue layer and sagittal abdominal diameter after both MICT and RT interventions. Similarly, fat mass decreased upon a MICT interventions by Oh et al. (42). In this latter study, no significant changes in body weight, visceral and subcutaneous adipose tissue area were observed upon HIIT. The 6-month MICT intervention of Eckard et al. (37) did not induce significant changes in body weight. In non-RCTs, there was a significant change in weight and BMI upon three month HIIT intervention in the study of Khaoshbaten et al. (52). In line, O’Gorman et al. (54) found a significant change in BMI and waist circumference upon HIIT. In two SIT interventions no effects on body weight and composition were seen (53, 56), albeit that a decrease in VAT mass was reported in the latter study (56).

**Figure 5:**
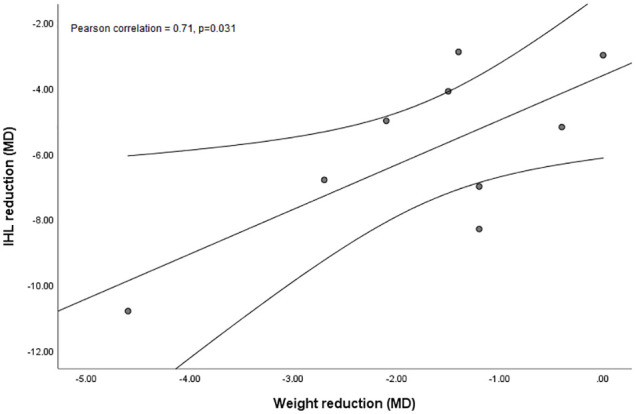
Simple scatter plot with linear fit line and 95% CI of intrahepatic lipids (IHL, %) reduction mean difference (MD) against weight reduction (kg; MD) upon aerobic exercise (AE) including both modalities, high-intensity interval training (HIIT) and moderate-intensity continuous training (MICT) interventions.

In order to study whether the significant reductions in weight are associated with the reductions in liver fat, we performed a Pearson correlation analysis with all studies. This showed that a reduction of IHL upon AE intervention correlated significantly with the weight reduction (r=0.714, p=0.031) (**Figure 5**). Of interest, reductions of IHL and weight were not correlated with the duration of the intervention or with any other measured parameter.

#### 3.4.3 Cardiorespiratory fitness

**Figure 6:**
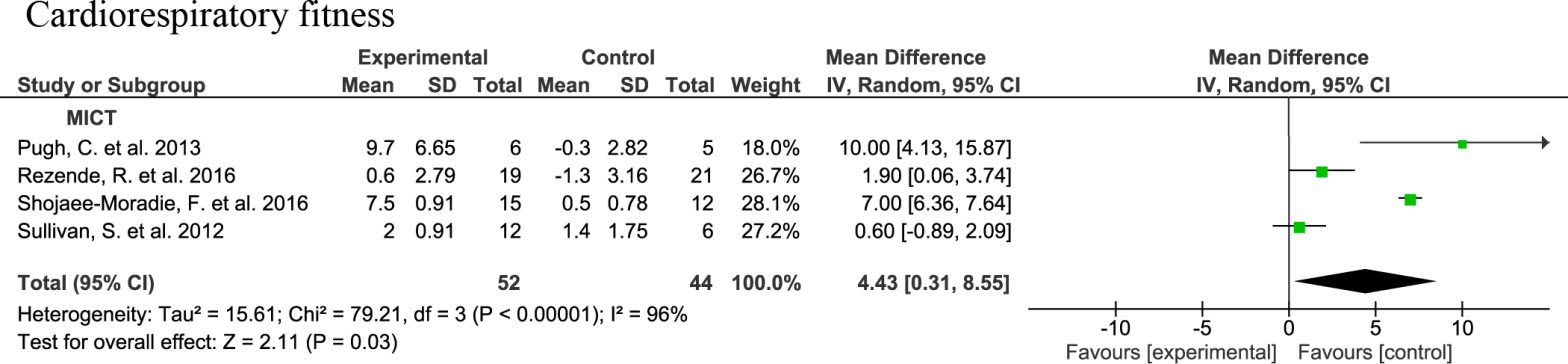
Forrest plot for the effect of moderate-intensity continuous training (MICT) on cardiorespiratory fitness level assessed as maximum or peak oxygen uptake (VO2max or VO2peak).

The meta-analysis for cardiorespiratory fitness was performed with MICT intervention studies since this outcome was not reported in the other interventions. Four studies assessing cardiorespiratory fitness either by VO2max (46) or VO2peak (43, 44, 47) upon 16- (43, 46, 47) or 24-week (44) MICT interventions are integrated in the meta-analysis. There is a significantly higher cardiorespiratory fitness in the MICT groups (n=52) compared to the controls (n=44) with a MD of 4.43 (95% CI: 0.31, 8.55, p=0.03) with a considerable heterogeneity (I2 = 96%, τ2 = 15.61, p<0.00001) (**Figure 6**).

In non-RCTs, a significant increase in VO2max was observed after a HIIT intervention of seven consecutive days (51). Also, VO2max is increased significantly after HIIT exercise, as well as when compared to the control group (54). There were significant increases in VO2peak and VO2max after SIT programs (51, 53).

In order to study whether the significant increase in cardiorespiratory fitness compared to controls are associated with the reductions in liver fat, we performed a Pearson correlation analysis with the studies. Cardiorespiratory fitness did not show significant correlation with liver fat (r=-0.04, p=0.98), or with liver transaminases (ALT r=88, p=0.32; AST r=0.94, p=0.22). Yet, these analyses were conducted with a very limited number of studies (n=3) due to the limited availability of the outcomes of interest and the independent variables.

#### 3.4.4 Glucose metabolism

**Figure 7:**
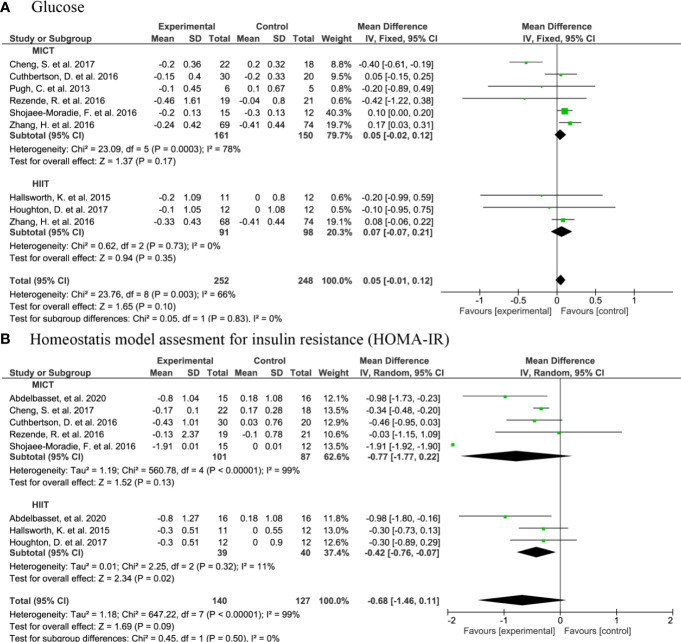
Forrest plot for the effect of moderate-intensity continuous training (MICT) and high-intensity interval training (HIIT) on: **(A)** glucose; **(B)** homeostatic Model Assessment for Insulin Resistance (HOMA-IR).

The meta analysis showed that there was no significant change in glucose concentrations upon MICT (MD: -0.04, 95% CI: -0.23, 0.15, n=161, p=0.70), HIIT (MD: 0.07, 95% CI: -0.07, 0.21, n=91, p=0.35), or AE in general (MD: -0.01, 95% CI: -0.15, 0.13, n=252, p=0.88) (**Figure 7A**). Yet, when compared to controls (n=40), HIIT subjects (n=39) had a lower HOMA-IR with a MD of -0.42 (95% CI: -0.76, -0.07, p=0.02) (**Figure 7B**).

Other proxies of glucose metabolism were investigated in a subset of the articles that were not part of the meta-analysis due to an incomparable study design. For instance, Bacchi et al. (33) reported a modest significant increase in the glucose disposal rate after AE intervention. Cevik et al. (34) did not show a significant change in glucose after the interventions. However, HOMA-IR was significantly decreased after AE with whole-body vibration but not in those without the vibration (34). Winn et al. (48) did not find a significant change in HOMA-IR upon HIIT or MICT. In non-RCTs, HOMA-IR decreased after a SIT program by Sergeant et al. (56), as well as by MacLean et al. (53). Haus et al. (51) reported a significant reduction in fasting plasma glucose concentrations after a 7-day HIIT.

#### 3.4.5 Plasma lipids

**Figure 8:**
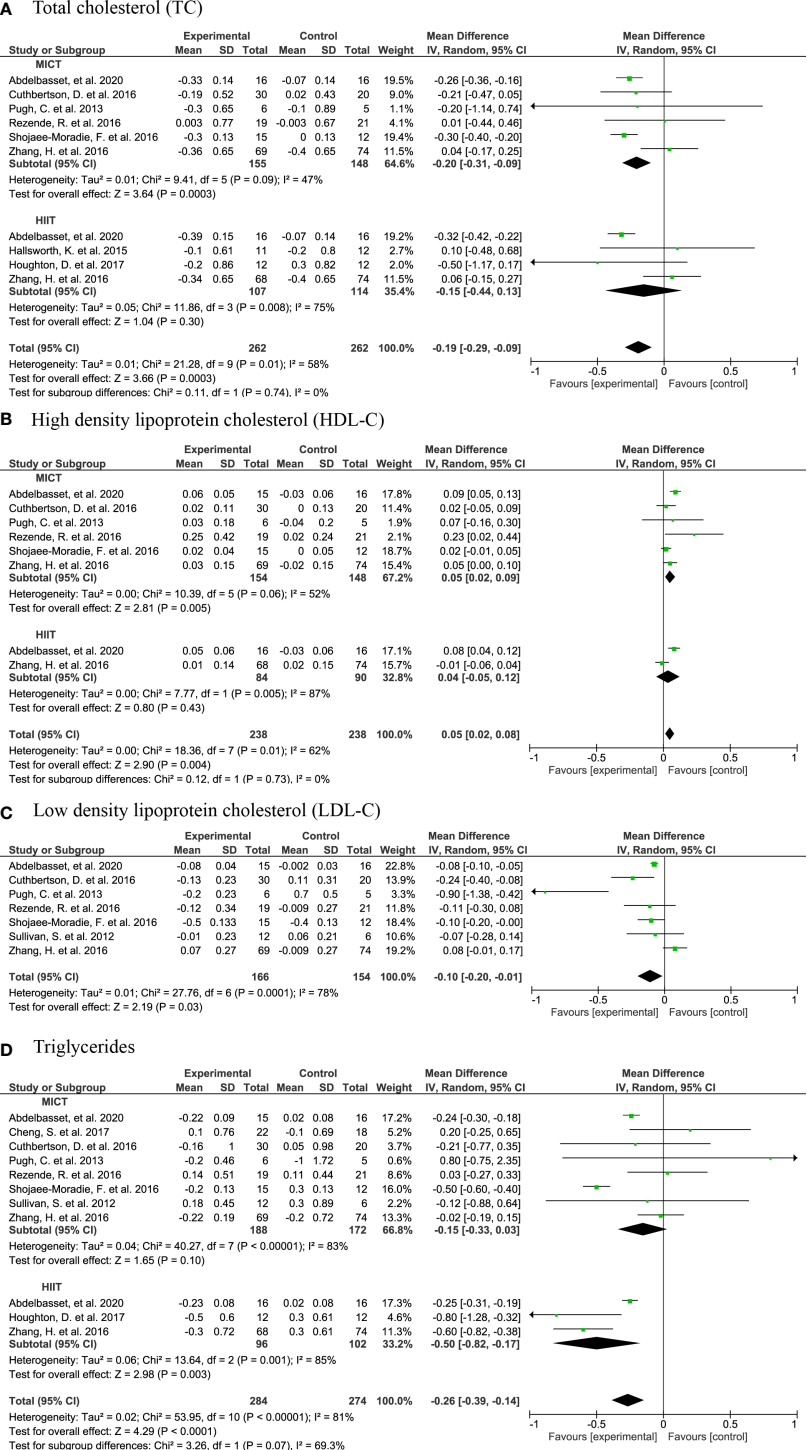
Forrest plot for the effect of moderate-intensity continuous training (MICT) and high-intensity interval training (HIIT) on plasma lipids: **(A)** total cholesterol (TC); **(B)** high-density lipoprotein cholesterol (HDL-C); **(C)** Forrest plot for the effect of MICT on low-density lipoprotein cholesterol (LDL-C); **(D)** triglycerides (TG).

In the meta analysis, the plasma total cholesterol concentration was significantly lower upon AE in general and upon MICT than in controls, with a MD of -0.19 (95% CI: -0.29, -0.09, n=261, p<0.001) and -0.20 (95% CI: -0.31, -0.09, n=154, p<0.001) (**Figure 8A**) for AE and MICT, respectively. In line, plasma LDL-C concentration was lower upon MICT, and HDL-C concentration was higher upon AE, MICT and HIIT, compared to controls (**Figures 8B, C**). When compared to controls, AE subjects had lower plasma TG concentrations (MD: -0.26, 95% CI: -0.39, -0.14, n=284, p<0.0001) with a considerable heterogeneity of 81% (τ2 = 0.02, p<0.0001) (**Figure 8D**).

The meta-analysis of lipid concentrations did not include the following studies due to non-comparable study designs. In the study of Winn et al. (48), plasma TC and HDL-C concentrations were unchanged upon MICT and HIIT (48). Bacchi et al. (33) and Cevik et al. (34) did not report a significant change in LDL-C after a HIIT intervention (34). Yet, plasma TG concentrations are decreased after MICT in the study by Bacchi et al. (33), but not in the study by Cevik et al. (34). In non-RCTs, there is a significant increase in plasma HDL-C concentrations upon a SIT (56). Also, Khaoshbaten et al. (52) observe a significant increase in HDL-C concentrations and a decrease in TG concentrations upon 3-month HIIT.

### 3.5 Sensitivity analysis

In order to examine the impact of the intervention duration on the meta-analyses, we conducted sensitivity analysis by removing Rezende et al. (n=19) (44) with a 24-week intervention, which did not lead to any significant changes. Furthermore, we performed a sensitivity analysis by removing Zhang et al. (n=135) (49) with a 26-week intervention from the analysis (**Table 3**). The analysis did not result in a significant difference in the IHL; however, the plasma AST results changed significantly before and after removing Zhang et al. (MD: -2.51, p=0.09 vs. MD: -4.43, p=0.002). In regard to the plasma TC, there was a significant change, after removing the data of Zhang et al. for HIIT (MD: -0.15 vs. -0.30) as well as for the HIIT for TG (MD: -0.50 vs. -0.47). After removing both Rezende et al. (44) and Zhang et al. (49), in addition to the significant changes mentioned by removing Zhang et al., TG for MICT changed significantly (MD: -0.15, p=0.10 vs. MD: -0.23, p=0.03) (**Table 4**).

**Table 3:** Results of meta-analyses before and after sensitivity analysis including mean difference (MD), 95% CI and significance level, as well as heterogeneity (I2) and significance level per intervention type, consisting of studies with similar intervention duration excluding study by Zhang et al.

Outcome	AE				MICT				HIIT			
	MD 95% CI	p-val	I2	p-val	MD 95% CI	p-val	I2	p-val	MD 95% CI	p-val	I2	p-val
IHL (%) Before	-4.10 [-5.33, -2.87]	<0.00001	35%	0.12	-5.19, [-7.33, -3.04]	<0.00001	45%	0.09	-3.41 [-4.74, -2.08]	<0.00001	10%	0.34
IHL (%) After	-4.08 [-5.77, -2.39]	<0.00001	43%	0.08	-5.92 [-9.01, -2.82]	0.0002	53%	0.06	-2.62 [-4.16, -1.08]	0.0009	0%	0.90
ALT (UI) Before	-3.78 [-5.58, -1.98]	<0.0001	44%	0.06	-3.59 [-5.60, -1.57]	0.0005	31%	0.19	-4.31 [-9.03, -.41]	0.07	65%	0.04
ALT (UI) After	-5.29 [-6.51, -4.08]	<0.00001	0%	0.78	-4.83 [-6.36, -3.30]	<0.00001	0%	0.92	-6.41 [-11.15, -1.66]	0.008	14%	0.31
AST (UI) Before	**-2.51** **[-5.41, 0.38]**	**0.09**	**83%**	**<0.00001**	-4.05 [-6.39, -1.71]	0.0007	63%	0.02	1.02 [-6.91, 8.94]	0.80	77%	0.01
AST (UI) After	**-4.43** **[-7.30, -1.57]**	**0.002**	**50%**	**0.06**	-5.16 [-6.67, -3.65]	<0.00001	8%	0.36	2.22 [-17.32, 21.76]	0.82	86%	0.008
Glucose (mmol/L) Before	0.05 [-0.01, 0.12]	0.10	66%	0.003	0.05 [-0.02, 0.12]	0.17	78%	0.0003	0.07 [-0.07, 0.21]	0.35	0%	0.73
Glucose (mmol/L) After	0.01 [-0.07, 0.09]	0.88	69%	0.003	0.01 [-0.07, 0.09]	0.82	79%	0.0007	-0.15 [-0.73, 0.42]	0.60	0%	0.87
TC (mmol/L) Before	-0.19 [-0.29, -0.09]	0.0003	58%	0.01	-0.20 [-0.31, -0.09]	0.0003	47%	0.09	**-0.15** **[-0.44, 0.13]**	**0.30**	**75%**	**0.008**
TC (mmol/L) After	-0.28 [-0.34, -0.23]	<0.00001	0%	0.68	-0.27 [-0.33, -0.20]	<0.00001	0%	0.72	**-0.30** **[-0.47, -0.12]**	**0.001**	**12%**	**0.32**
LDL-C (mmol/L) Before	–	–	–	–	-0.10 [-0.20, -0.01]	0.03	78%	0.0001	–	–	–	–
LDL-C (mmol/L) After	–	–	–	–	-0.14 [-0.24, -0.05]	0.003	68%	0.009	–	–	–	–
HDL-C (mmol/L) Before	0.05 [0.02, 0.08]	0.0004	62%	0.01	0.05 [0.02, 0.09]	0.005	52%	0.06	0.04 [-0.05, 0.12]	0.004	62%	0.001
HDL-C (mmol/L) After	0.06 [0.02, 0.10]	0.002	58%	0.04	0.06 [0.01, 0.11]	0.03	61%	0.03	0.08 [0.04, 0.12]	0.0002	NA	NA
Triglycerides (mmol/L) Before	-0.26 [-0.39, -0.14]	<0.0001	81%	<0.00001	-0.15 [-0.33, 0.03]	0.10	83%	<0.00001	**-0.50** **[-0.82, -0.17]**	**0.003**	**85%**	**0.001**
Triglycerides (mmol/L) After	-0.26 [-0.39, -0.14]	<0.0001	78%	<0.00001	-0.19 [-0.38, 0.01]	0.07	81%	<0.0001	**-0.47** **[-1.00, 0.06]**	**0.08**	**80%**	**0.03**
Weight (kg) Before	-1.90 [-2.46, -1.35]	<0.00001	0%	0.83	-1.80 [-2.52, -1.09]	<0.00001	0%	0.59	-2.06 [-2.93, -1.18]	<0.00001	0%	0.83
Weight (kg) After	-2.26 [-3.43, -1.08]	0.0002	0%	0.82	-2.35 [-3.57, -1.14]	0.0001	0%	0.65	-0.80 [-5.53, 3.94]	0.74	0%	0.77

ALT, alanine transferase; AST, aspartate transferase; HIIT, high-intensity interval training; HOMA-IR, homeostasic assessment for insulin resistance; HDL-C, high density lipoprotein cholesterol; IHL, intrahepatic lipids; kg, kilogram; LDL-C, low density lipoprotein cholesterol; MICT, moderate-intensity continuous training; mmol/l, milligram per deciliter; UI, international unit; VO2max, maximum oxygen consumption. NA; not applicable. Results of sensitivity analyses that differ in significance from the original meta-analysis are marked in bold.

**Table 4:** Results of meta-analyses before and after sensitivity analysis including mean difference (MD), 95% CI and significance level, as well as heterogeneity (I2) and significance level per intervention type, consisting of studies with similar intervention duration excluding study by Zhang et al. and Rezende et al.

Outcome	AE				MICT				HIIT			
	MD 95% CI	p-val	I2	p-val	MD 95% CI	p-val	I2	p-val	MD 95% CI	p-val	I2	p-val
IHL (%) Before	-4.10 [-5.33, -2.87]	<0.00001	35%	0.12	-5.19, [-7.33, -3.04]	<0.00001	45%	0.09	-3.41 [-4.74, -2.08]	<0.00001	10%	0.34
IHL (%) After	-4.08 [-5.77, -2.39]	<0.00001	43%	0.08	-5.92 [-9.01, -2.82]	0.0002	53%	0.06	-2.62 [-4.16, -1.08]	0.0009	0%	0.90
ALT (UI) Before	-3.78 [-5.58, -1.98]	<0.0001	44%	0.06	-3.59 [-5.60, -1.57]	0.0005	31%	0.19	-4.31 [-9.03, -.41]	0.07	65%	0.04
ALT (UI) After	-5.30 [-6.52, -4.08]	<0.00001	0%	0.69	-4.84 [-6.37, -3.30]	<0.00001	0%	0.83	-6.41 [-11.15, -1.66]	0.008	14%	0.31
AST (UI) Before	**-2.51** **[-5.41, 0.38]**	**0.09**	**83%**	**<0.00001**	-4.05 [-6.39, -1.71]	0.0007	63%	0.02	1.02 [-6.91, 8.94]	0.80	77%	0.01
AST (UI) After	**-4.91** **[-7.69, -2.12]**	**0.0005**	**50%**	**0.08**	-5.17 [-6.40, -3.95]	<0.00001	0%	0.53	2.22 [-17.32, 21.76]	0.82	86%	0.008
Glucose (mmol/L) Before	0.05 [-0.01, 0.12]	0.10	66%	0.003	0.05 [-0.02, 0.12]	0.17	78%	0.0003	0.07 [-0.07, 0.21]	0.35	0%	0.73
Glucose (mmol/L) After	0.01 [-0.07, 0.09]	0.80	73%	0.002	0.01 [-0.07, 0.09]	0.74	84%	0.0004	-0.15 [-0.73, 0.42]	0.60	0%	0.87
TC (mmol/L) Before	-0.19 [-0.29, -0.09]	0.0003	58%	0.01	-0.20 [-0.31, -0.09]	0.0003	47%	0.09	**-0.15** **[-0.44, 0.13]**	**0.30**	**75%**	**0.008**
TC (mmol/L) After	-0.29 [-0.34, -0.23]	<0.00001	0%	0.78	-0.27 [-0.34, -0.21]	<0.00001	0%	0.90	**-0.30** **[-0.47, -0.12]**	**0.001**	**12%**	**0.32**
LDL-C (mmol/L) Before	–	–	–	–	-0.10 [-0.20, -0.01]	0.03	78%	0.0001	–	–	–	–
LDL-C (mmol/L) After	–	–	–	–	-0.15 [-0.27, -0.04]	0.007	74%	0.004	–	–	–	–
HDL-C (mmol/L) Before	0.05 [0.02, 0.08]	0.0004	62%	0.01	0.05 [0.02, 0.09]	0.005	52%	0.06	0.04 [-0.05, 0.12]	0.004	62%	0.001
HDL-C (mmol/L) After	0.06 [0.02, 0.09]	0.002	57%	0.05	0.05 [0.00, 0.09]	0.05	61%	0.05	0.08 [0.04, 0.12]	0.0002	NA	NA
Triglycerides (mmol/L) Before	-0.26 [-0.39, -0.14]	<0.0001	81%	<0.00001	**-0.15** **[-0.33, 0.03]**	**0.10**	**83%**	**<0.00001**	**-0.50** **[-0.82, -0.17]**	**0.003**	**85%**	**0.001**
Triglycerides (mmol/L) After	-0.30 [-0.43, -0.17]	<0.0001	79%	<0.0001	**-0.23** **[-0.45, -0.02]**	**0.03**	**81%**	**<0.0001**	**-0.47** **[-1.00, 0.06]**	**0.08**	**80%**	**0.03**
Weight (kg) Before	-1.90 [-2.46, -1.35]	<0.00001	0%	0.83	-1.80 [-2.52, -1.09]	<0.00001	0%	0.59	-2.06 [-2.93, -1.18]	<0.00001	0%	0.83
Weight (kg) After	-2.26 [-3.43, -1.08]	0.0002	0%	0.82	-2.35 [-3.57, -1.14]	0.0001	0%	0.65	-0.80 [-5.53, 3.94]	0.74	0%	0.77

ALT, alanine transferase; AST, aspartate transferase; HIIT, high-intensity interval training; HOMA-IR, homeostasic assessment for insulin resistance; HDL-C, high density lipoprotein cholesterol; IHL, intrahepatic lipids; kg, kilogram; LDL-C, low density lipoprotein cholesterol; MICT, moderate-intensity continuous training; mmol/l, milligram per deciliter; UI, international unit; VO2max, maximum oxygen consumption. NA; not applicable. Results of sensitivity analyses that differ in significance from the original meta-analysis are marked in bold.

### 3.6 Quality assessment

**Figure 9:**
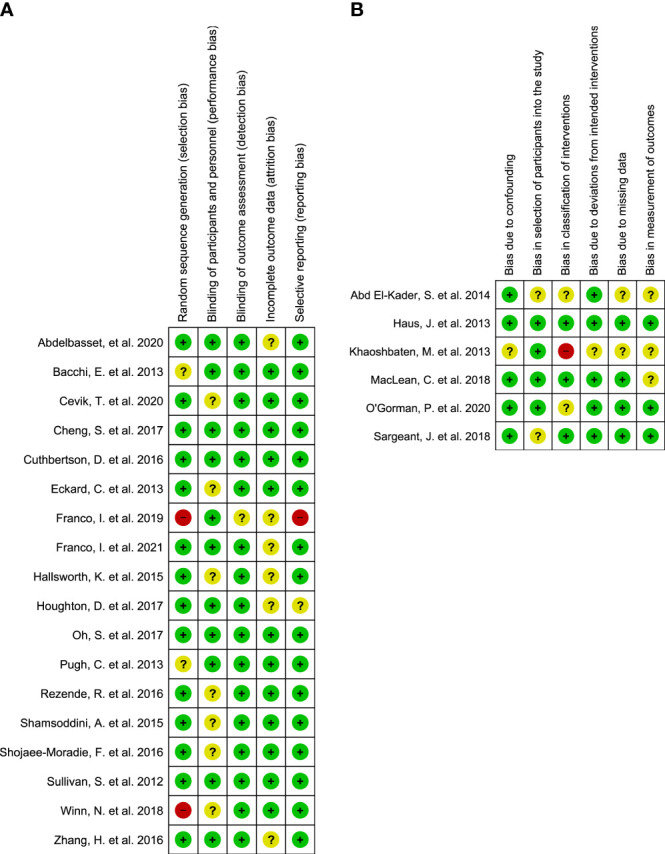
The results of the quality assessment of **(A)** randomized controlled trials (RCTs); **(B)** non-randomized controlled trials (non-RCTs).

The quality assessment resulted in overall low risk of bias in all domains for three RCTs (35, 42, 47), while ten articles showed some concerns in the overall judgment since they had a moderate risk in one domain (32–34, 37, 39, 43–46, 49) (**Figure 9A**). One study showed a high risk of classification of interventions as the intervention groups were not clearly defined, also the information used to define the intervention groups was not mentioned at the start (52). Two studies were scored high risk of selection bias, since they did not use random sequence (38, 48). Studies that raised concerns in the quality are included in the narrative review, however, were not integrated in the meta-analysis. Quality assessment of non-RCTs is shown in **Figure 9B**. Non-RCTs are included in the narrative review.

## 4 Discussion

This systematic review assessed the effects of two types of AE (MICT and HIIT) without dietary changes on NAFLD and related metabolic parameters. Our findings showed that both HIIT and MICT significantly reduced IHL, an effect significantly associated with reductions in body weight. In addition, MICT but not HIIT reduced liver transaminases and both AE types improved the plasma lipid profile. Although AE did not affect blood glucose concentrations, HIIT improved HOMA-IR.

In general, we observed no effect of AE on fibrosis, related varying results on liver stiffness. While MICT did not lead to improvements in liver fibrosis in the included studies (34, 44), HIIT did significantly improve liver fibrosis (42, 54). Previously, fibrosis regression has been associated with a dose-dependent increase in physical activity (51). In line, insufficient physical activity is an independent predictor of fibrosis in NAFLD (21). Despite the fact that liver fibrosis is the most important determinant of the risk of liver-related mortality (57), most studies invest in detecting steatosis and not fibrosis. Like fibrosis, liver inflammation as an endpoint in lifestyle interventions has been overlooked. In contrast, plasma concentrations of the liver enzymes AST, ALT and γGT as proxy of liver functioning are often measured. Increased plasma concentrations of these liver enzymes are associated with an increased risk for the progression to advanced liver disease (58), characterized by inflammation and fibrosis. Yet, non-invasive NAFLD tests that often include the plasma transaminase concentrations are not optimal (59). Thus, in order to more firmly draw conclusions on the effects of exercise on NASH and liver fibrosis, well controlled exercise intervention studies with sufficient sample sizes and elucidative endpoints in patients with advanced stages of NAFLD are called for.

Nearly all included studies showed lower IHL after MICT and HIIT. This is in line with a recent systematic review in which the effects of MICT and HIIT were studied in a wide range of patients, i.e., those with obesity and T2DM, without information on NAFLD (60). We found a significant positive correlation between the reductions in body weight and IHL upon AE. Of interest, it has already been suggested that effects of AE on IHL might be mediated by alterations in body composition, e.g., loss of body weight and adipose tissue mass, or an increased muscle mass (61). In line, another meta-analysis reported similar association of body weight loss and decrease in IHL (62). Based on these earlier studies and our present results, we can conclude that AE-induced weight loss and changes in body composition are crucial in reducing IHL. It also underscores that weight loss *per se* is beneficial for the liver.

We did not detect a differential effect of MICT and HIIT on IHL. This is in line with previous studies in which HIIT and MICT were compared (32, 36, 42, 48), underscoring that neither was superior in ameliorating steatosis. However, some studies reported that moderate-intensity training is better in reducing liver fat than low-intensity training (63). This notion was supported by a prospective study in which vigorous but not moderate physical activity lowered the risk for NAFLD (64). Yet, current guidelines state that any level of physical activity or exercise can be beneficial for patients with NAFLD (17). Perhaps, practically, the paramount aim may be to engage sedentary patients to increase their activity at any level (22). In order to increase the engagement for long-term adherence to exercise, physical activity and exercise programs should be personalized according to the limitations and preferences (i.e. comorbidities, clinical characteristics, personal goals) of the individual patient with NAFLD (22, 54, 65). More studies need to investigate the different modalities of AE in patients with different stages of NAFLD to direct these personalized guidelines.

We did not find a significant change in blood glucose concentrations upon AE, which was in line with a previous study (66). Yet, a modest, but a significant decrease was found in HOMA-IR upon HIIT. Patients with NASH often have a higher HOMA-IR than healthy controls (67) and thus, are insulin resistant (68). Insulin resistance plays a central role in hepatic lipid accumulation (69). Consequently, lipid metabolism is also often altered in patients with NAFLD (70), and therefore patients with NAFLD present an increased asCVD related mortality (5). Evidently, elevated LDL-C concentrations, promoting the development of thrombus and plaque (71, 72), and elevated concentrations of plasma TG (73) increase the risk of asCVD. Simultaneously, an increase of HDL-C concentrations is associated with a significant risk reduction (7, 71). Our meta-analysis showed significant higher plasma HDL-C concentrations and lower plasma LDL-C and TC concentrations upon the MICT compared to controls. Plasma TG concentrations were only lower upon HIIT compared to controls. Another systematic review about AE-induced changes in NAFLD, however, did not report significant changes in plasma lipids, except for a reduction in plasma TG concentrations (66). Physical activity is associated with lower asCVD related mortality in patients with NAFLD (21), which is probably partly due to the improvements in lipid profile, and partly due to improved cardiorespiratory fitness (74). In fact, we observed higher cardiorespiratory fitness upon MICT compared to control in our meta-analyses. These metabolic targets, cardiorespiratory fitness together with plasma lipid profile by exercise should be viewed as part of holistic treatment of patients with NAFLD who present an increased risk for asCVD.

Most of the included studies performed supervised exercise sessions, regardless of the modality (32, 33, 35–37, 40, 42, 44–47, 49, 51, 54, 56). In addition to the supervised sessions, some of the studies prescribed home-based/unsupervised training to the study subjects (40, 54, 75). Yet, this does have a major impact to our meta-analyses. The compliance to the exercise intervention was determined either by cardiopulmonary exercise test (43, 46–48, 54, 56, 75) or by physical activity accelerometer (45, 47, 75) or/and questionnaire (40, 75). Given that limited number of studies determined the cardiorespiratory fitness at the follow-up, the meta-analysis for cardiorespiratory fitness could be only conducted with four comparable studies. In future studies, it would be ideal to assess the compliance to the intervention by the exercise test for reliable results, and simultaneously, study the efficacy of the aerobic exercise intervention.

Regarding limitations, lack of sufficient data impeded conducting meta-analysis for all interventions and outcomes, while relatively small number of studies were included in each conducted meta-analysis. In addition, the small number of studies precluded the detection of publication bias on a funnel plot. Publication bias may also arise from the grey literature that the systematic review of the literature do not consider. To minimize the risk of publication bias, we used a comprehensive and sensitive search strategy for finding published full texts and conference abstracts as well as backward reference searching of the included studies. Furthermore, we detect variable degrees of heterogeneity in the meta-analyses. In general, meta-analyses with high heterogeneity should be interpreted cautiously. High heterogeneity may be driven from statistical and methodological heterogeneity since clinical heterogeneity has been kept minimum by including well characterized patient group and interventions (31). Different methods that an outcome is measured may lead to differential intervention effect sizes resulting in a high heterogeneity, for example the meta-analyses of HOMA-IR have high heterogeneity. HOMA-IR was calculated by using the reported values of glucose and insulin that in the first place are measured in different laboratories, possibly by different methods, leading to high heterogeneity in the meta-analyses.

In conclusion, the evidence available for exercise interventions in patients with NAFLD clearly indicates that hepatic fat is decreased by AE. It can be mediated by reduction of body weight, regardless of exercise modality, with a concomitant decrease of liver enzymes upon MICT, and improvements in plasma lipids. In addition, HIIT may improve HOMA-IR. Yet, there is a striking lack of studies with liver histology, precluding any firm conclusions pertaining to the effect of AE on NASH or liver fibrosis, the main clinically relevant disease parameters in NAFLD. In order to draw conclusions on the effects of exercise on NASH and liver fibrosis and determine the optimal modality of AE, well-controlled exercise intervention studies with elucidative endpoints in patients with advanced stages of NAFLD are called for.

## Data availability statement

Publicly available datasets were analyzed in this study.

## Author contributions

VH and JB contributed equally to the protocol of the systematic review, and design and organization of the manuscript, as well as writing, reviewing, editing of the manuscript. VH and JB performed together the quality assessment, data extraction, statistical analysis, and meta-analysis. YV contributed to the protocol of the systematic review as well as reviewing and editing of the manuscript. JD designed and conducted the search. AG, MN, and JD contributed to reviewing and editing of the manuscript. AH supervised the work and contributed to the protocol of the systematic review, as well as reviewing and editing of the manuscript. All authors contributed to the article and approved the submitted version.

## Funding

This work has received funding from an ITN Marie Curie BestTreat – Building a Gut Microbiome Engineering Toolbox for *In-Situ* Therapeutic Treatments for Non-alcoholic Fatty Liver Disease No. 813781 ITN BestTreat (on which VH is appointed). AH was supported by the Amsterdam UMC Fellowship, Health–Holland TKI-PPP grants, grants for the Dutch Gastroenterology Foundation and by a research grant from Novo Nordisk. MN was supported by a personal ZONMW VICI grant 2020 [09150182010020].

## Conflict of interest

The authors declare that the research was conducted in the absence of any commercial or financial relationships that could be construed as a potential conflict of interest.

## Publisher’s note

All claims expressed in this article are solely those of the authors and do not necessarily represent those of their affiliated organizations, or those of the publisher, the editors and the reviewers. Any product that may be evaluated in this article, or claim that may be made by its manufacturer, is not guaranteed or endorsed by the publisher.

## References

[1] Perumpail BJ Khan MA Yoo ER Cholankeril G Kim D Ahmed A . Clinical epidemiology and disease burden of nonalcoholic fatty liver disease. World J Gastroenterol (2017) 23(47):8263–76. doi: 10.3748/wjg.v23.i47.8263 PMC5743497 29307986

[2] Bluher M . Obesity: global epidemiology and pathogenesis. Nat Rev Endocrinol (2019) 15(5):288–98. doi: 10.1038/s41574-019-0176-8 30814686

[3] Ress C Kaser S . Mechanisms of intrahepatic triglyceride accumulation. World J Gastroenterol (2016) 22(4):1664–73. doi: 10.3748/wjg.v22.i4.1664 PMC4721997 26819531

[4] Mohamed RZ Jalaludin MY Anuar Zaini A . Predictors of non-alcoholic fatty liver disease (NAFLD) among children with obesity. J Pediatr Endocrinol Metab (2020) 33(2):247–53. doi: 10.1515/jpem-2019-0403 31926095

[5] Golabi P Fukui N Paik J Sayiner M Mishra A Younossi ZM . Mortality risk detected by atherosclerotic cardiovascular disease score in patients with nonalcoholic fatty liver disease. Hepatol Commun (2019) 3(8):1050–60. doi: 10.1002/hep4.1387 PMC6671783 31388626

[6] Younossi Z Anstee QM Marietti M Hardy T Henry L Eslam M . Global burden of NAFLD and NASH: trends, predictions, risk factors and prevention. Nat Rev Gastroenterol Hepatol (2018) 15(1):11–20. doi: 10.1038/nrgastro.2017.109 28930295

[7] Ahmed HM Miller M Nasir K McEvoy JW Herrington D Blumenthal RS . Primary low level of high-density lipoprotein cholesterol and risks of coronary heart disease, cardiovascular disease, and death: Results from the multi-ethnic study of atherosclerosis. Am J Epidemiol (2016) 183(10):875–83. doi: 10.1093/aje/kwv305 PMC4867155 27189327

[8] Taylor RS Taylor RJ Bayliss S Hagström H Nasr P Schattenberg JM . Association between fibrosis stage and outcomes of patients with nonalcoholic fatty liver disease: A systematic review and meta-analysis. Gastroenterology (2020) 158(6):1611–25.e12. doi: 10.1053/j.gastro.2020.01.043 32027911

[9] Dulai PS Singh S Patel J Soni M Prokop LJ Younossi Z . Increased risk of mortality by fibrosis stage in nonalcoholic fatty liver disease: Systematic review and meta-analysis. Hepatology (2017) 65(5):1557–65. doi: 10.1002/hep.29085 PMC5397356 28130788

[10] Amarapurka DN Amarapurkar AD Patel ND Agal S Baigal R Gupte P . Nonalcoholic steatohepatitis (NASH) with diabetes: predictors of liver fibrosis. Ann Hepatol (2006) 5(1):30–3. doi: 10.1016/S1665-2681(19)32036-8 16531962

[11] Zhu L Baker SS Gill C Liu W Alkhouri R Baker RD . Characterization of gut microbiomes in nonalcoholic steatohepatitis (NASH) patients: a connection between endogenous alcohol and NASH. Hepatology (2013) 57(2):601–9. doi: 10.1002/hep.26093 23055155

[12] Byrne CD Targher G . NAFLD: a multisystem disease. J Hepatol (2015) 62(1 Suppl):S47–64. doi: 10.1016/j.jhep.2014.12.012 25920090

[13] Marchesini G Brizi M Morselli-Labate AM Bianchi G Bugianesi E McCullough AJ . Association of nonalcoholic fatty liver disease with insulin resistance. Am J Med (1999) 107(5):450–5. doi: 10.1016/S0002-9343(99)00271-5 10569299

[14] Sheldon GF Peterson SR Sanders R . Hepatic dysfunction during hyperalimentation. Arch Surg (1978) 113(4):504–8. doi: 10.1001/archsurg.1978.01370160162028 416812

[15] Meroni M Longo M Tria G Dongiovanni P . Genetics is of the essence to face NAFLD. Biomedicines (2021) 9(10):1359. doi: 10.3390/biomedicines9101359 34680476 PMC8533437

[16] Loomba R Friedman SL Shulman GI . Mechanisms and disease consequences of nonalcoholic fatty liver disease. Cell (2021) 184(10):2537–64. doi: 10.1016/j.cell.2021.04.015 PMC12168897 33989548

[17] European Association for the Study of the Liver (EASL) European Association for the Study of Diabetes (EASD) European Association for the Study of Obesity (EASO) . EASL-EASD-EASO clinical practice guidelines for the management of non-alcoholic fatty liver disease. J Hepatol (2016) 64(6):1388–402. doi: 10.1016/j.jhep.2015.11.004 27062661

[18] Younossi ZM Corey KE Lim JK . AGA clinical practice update on lifestyle modification using diet and exercise to achieve weight loss in the management of nonalcoholic fatty liver disease: Expert review. Gastroenterology (2021) 160(3):912–8. doi: 10.1053/j.gastro.2020.11.051 33307021

[19] Chalasani N Younossi Z Lavine JE Charlton M Cusi K Rinella M . The diagnosis and management of nonalcoholic fatty liver disease: Practice guidance from the American association for the study of liver diseases. Hepatology (2018) 67(1):328–57. doi: 10.1002/hep.29367 28714183

[20] Patel H Alkhawam H Madanieh R Shah N Kosmas CE Vittorio TJ . Aerobic vs anaerobic exercise training effects on the cardiovascular system. World J Cardiol (2017) 9(2):134–8. doi: 10.4330/wjc.v9.i2.134 PMC5329739 28289526

[21] Kim D Konyn P Cholankeril G Ahmed A . Physical activity is associated with nonalcoholic fatty liver disease and significant fibrosis measured by FibroScan. Clin Gastroenterol Hepatol (2021) 20(6):e1438–e1455. doi: 10.1016/j.cgh.2021.06.029 34214678

[22] Hallsworth K Adams LA . Lifestyle modification in NAFLD/NASH: Facts and figures. JHEP Rep (2019) 1(6):468–79. doi: 10.1016/j.jhepr.2019.10.008 PMC7005657 32039399

[23] Berger D Desai V Janardhan S . Con: Liver biopsy remains the gold standard to evaluate fibrosis in patients with nonalcoholic fatty liver disease. Clin Liver Dis (2019) 13(4):114–6. doi: 10.1002/cld.740 PMC6491029 31061705

[24] Page MJ McKenzie JE Bossuyt PM Boutron I Hoffmann TC Mulrow CD . The PRISMA 2020 statement: an updated guideline for reporting systematic reviews. Bmj (2021) 372:n71. doi: 10.1136/bmj.n71 33782057 PMC8005924

[25] Houttu VBJ Vali J Daams J Grefhorst A Nieuwdorp M Holleboom AG . Can aerobic exercise reduce NASH and liver fibrosis in patients with non-alcoholic fatty liver disease? a systematic review of the literature and meta-analysis PROSPERO CRD42021270059: International prospective register of systematic reviews (2022). Available at: https://www.crd.york.ac.uk/prospero/#myprospero. 10.3389/fendo.2022.1032164 PMC9669057 36407307

[26] Ouzzani M Hammady H Fedorowicz Z Elmagarmid A . Rayyan-a web and mobile app for systematic reviews. Syst Rev (2016) 5(1):210. doi: 10.1186/s13643-016-0384-4 27919275 PMC5139140

[27] Sterne JAC SJ Page MJ Elbers RG Blencowe NS Boutron I Cates CJ . RoB 2: a revised tool for assessing risk of bias in randomised trials. BMJ (2019) 28(366):l4898. doi: 10.1136/bmj.l4898 31462531

[28] Sterne JAC HM Reeves BC Savović J Berkman ND Viswanathan M Henry D . ROBINS-I: a tool for assessing risk of bias in non-randomized studies of interventions. BMJ (2016) 355:i4919. doi: 10.1136/bmj.i4919 27733354 PMC5062054

[29] Higgins JPT Thomas J Chandler J Cumpston M Li T Page MJ Welch VA . Cochrane handbook for systematic reviews of interventions version 6.3 (updated February 2022). Cochrane (2022). Available at: www.training.cochrane.org/handbook.

[30] The Cochrane Collaboration . Review manager web (RevMan web). (2020) 2020, RW.

[31] Deeks JJ HJ Altman DG . Chapter 10: Analysing data and undertaking meta-analyses. In: Higgins JPT Thomas J Chandler J Cumpston M Li T Page MJ Welch VA , editors. Cochrane handbook for systematic reviews of interventions version 6.3 (updated February 2022). Cochrane (2022). Available at: www.training.cochrane.org/handbook.

[32] Abdelbasset WK Tantawy SA Kamel DM Alqahtani BA Soliman GS . A randomized controlled trial on the effectiveness of 8-week high-intensity interval exercise on intrahepatic triglycerides, visceral lipids, and health-related quality of life in diabetic obese patients with nonalcoholic fatty liver disease. Medicine (2019) 98(12):e14918. doi: 10.1097/MD.0000000000014918 30896648 PMC6708753

[33] Bacchi E Negri C Targher G Faccioli N Lanza M Zoppini G . Both resistance training and aerobic training reduce hepatic fat content in type 2 diabetic subjects with nonalcoholic fatty liver disease (the RAED2 Randomized Trial). Hepatology (2013) 58(4):1287–95. doi: 10.1002/hep.26393 23504926

[34] Çevik Saldiran T Mutluay FK Yağci İ Yilmaz Y . Impact of aerobic training with and without whole-body vibration training on metabolic features and quality of life in non-alcoholic fatty liver disease patients. Ann Endocrinol (2020) 81(5):493–9. doi: 10.1016/j.ando.2020.05.003 32768394

[35] Cheng S Ge J Zhao C Le S Yang Y Ke D . Effect of aerobic exercise and diet on liver fat in pre-diabetic patients with non-alcoholic-fatty-liver-disease: A randomized controlled trial. Sci Rep (2017) 7(1):15952. doi: 10.1038/s41598-017-16159-x 29162875 PMC5698376

[36] Cuthbertson DJ Shojaee-Moradie F Sprung VS Jones H Pugh CJ Richardson P . Dissociation between exercise-induced reduction in liver fat and changes in hepatic and peripheral glucose homoeostasis in obese patients with non-alcoholic fatty liver disease. Clin Sci (2016) 130(2):93–104. doi: 10.1042/CS20150447 26424731

[37] Eckard C Cole R Lockwood J Torres DM Williams CD Shaw JC . Prospective histopathologic evaluation of lifestyle modification in nonalcoholic fatty liver disease: a randomized trial. Therap Adv Gastroenterol (2013) 6(4):249–59. doi: 10.1177/1756283X13484078 PMC3667474 23814606

[38] Franco I Bianco A Diaz MDP Bonfiglio C Chiloiro M Pou SA . Effectiveness of two physical activity programs on non-alcoholic fatty liver disease. a randomized controlled clinical trial. Rev Fac Cien Med Univ Nac Cordoba (2019) 76(1):26–36. doi: 10.31053/1853.0605.v76.n1.21638 30882339

[39] Franco I Bianco A Mirizzi A Campanella A Bonfiglio C Sorino P . Physical activity and low glycemic index Mediterranean diet: Main and modification effects on NAFLD score. results from a randomized clinical trial. Nutrients (2020) 13(1):66. doi: 10.3390/nu13010066 PMC7823843 33379253

[40] Hallsworth K Thoma C Hollingsworth KG Cassidy S Anstee QM Day CP . Modified high-intensity interval training reduces liver fat and improves cardiac function in non-alcoholic fatty liver disease: a randomized controlled trial. Clin Sci (Lond) (2015) 129(12):1097–105. doi: 10.1042/CS20150308 26265792

[41] Houghton D Thoma C Hallsworth K Cassidy S Hardy T Burt AD . Exercise reduces liver lipids and visceral adiposity in patients with nonalcoholic steatohepatitis in a randomized controlled trial. Clin Gastroenterol Hepatol (2017) 15(1):96–102.e3. doi: 10.1016/j.cgh.2016.07.031 27521509 PMC5196006

[42] Oh S So R Shida T Matsuo T Kim B Akiyama K . High-intensity aerobic exercise improves both hepatic fat content and stiffness in sedentary obese men with nonalcoholic fatty liver disease. Sci Rep (2017) 7:43029. doi: 10.1038/srep43029 28223710 PMC5320441

[43] Pugh CJ Cuthbertson DJ Sprung VS Kemp GJ Richardson P Umpleby AM . Exercise training improves cutaneous microvascular function in nonalcoholic fatty liver disease. Am J Physiol Endocrinol Metab (2013) 305(1):E50–8. doi: 10.1152/ajpendo.00055.2013 23651847

[44] Rezende RE Duarte SM Stefano JT Roschel H Gualano B de Sa Pinto AL . Randomized clinical trial: benefits of aerobic physical activity for 24 weeks in postmenopausal women with nonalcoholic fatty liver disease. Menopause (2016) 23(8):876–83. doi: 10.1097/GME.0000000000000647 27458060

[45] Shamsoddini A Sobhani V Ghamar Chehreh ME Alavian SM Zaree A . Effect of aerobic and resistance exercise training on liver enzymes and hepatic fat in Iranian men with nonalcoholic fatty liver disease. Hepat Mon (2015) 15(10):e31434. doi: 10.5812/hepatmon.31434 26587039 PMC4644631

[46] Shojaee-Moradie F Cuthbertson DJ Barrett M Jackson NC Herring R Thomas EL . Exercise training reduces liver fat and increases rates of VLDL clearance but not VLDL production in NAFLD. J Clin Endocrinol Metab (2016) 101(11):4219–28. doi: 10.1210/jc.2016-2353 27583475

[47] Sullivan S Kirk EP Mittendorfer B Patterson BW Klein S . Randomized trial of exercise effect on intrahepatic triglyceride content and lipid kinetics in nonalcoholic fatty liver disease. Hepatology (2012) 55(6):1738–45. doi: 10.1002/hep.25548 PMC3337888 22213436

[48] Winn NC Liu Y Rector RS Parks EJ Ibdah JA Kanaley JA . Energy-matched moderate and high intensity exercise training improves nonalcoholic fatty liver disease risk independent of changes in body mass or abdominal adiposity - a randomized trial. Metabolism (2018) 78:128–40. doi: 10.1016/j.metabol.2017.08.012 28941598

[49] Zhang HJ He J Pan LL Ma ZM Han CK Chen CS . Effects of moderate and vigorous exercise on nonalcoholic fatty liver disease: A randomized clinical trial. JAMA Intern Med (2016) 176(8):1074–82. doi: 10.1001/jamainternmed.2016.3202 27379904

[50] Abd El-Kader SM Al-Jiffri OH Al-Shreef FM . Markers of liver function and inflammatory cytokines modulation by aerobic versus resisted exercise training for nonalcoholic steatohepatitis patients. Afr Health Sci (2014) 14(3):551–7. doi: 10.4314/ahs.v14i3.8 PMC4209634 25352871

[51] Haus JM Solomon TP Kelly KR Fealy CE Kullman EL Scelsi AR . Improved hepatic lipid composition following short-term exercise in nonalcoholic fatty liver disease. J Clin Endocrinol Metab (2013) 98(7):E1181–8. doi: 10.1210/jc.2013-1229 PMC3701282 23616151

[52] Khaoshbaten M Gholami N Sokhtehzari S Monazami AH Nejad MR . The effect of an aerobic exercise on serum level of liver enzymes and liver echogenicity in patients with non alcoholic fatty liver disease. Gastroenterol Hepatol Bed Bench (2013) 6(Suppl 1):S112–6. doi: 10.22037/ghfbb.v6i0.482 PMC4017540 24834280

[53] MacLean C Dillon J Babraj JA Vollaard NBJ . The effect of low volume sprint interval training in patients with non-alcoholic fatty liver disease. Physician Sportsmedicine (2018) 46(1):87–92. doi: 10.1080/00913847.2018.1411171 29183220

[54] O'Gorman P Naimimohasses S Monaghan A Kennedy M Melo AM NF D . Improvement in histological endpoints of MAFLD following a 12-week aerobic exercise intervention. Aliment Pharmacol Ther (2020) 52(8):1387–98. doi: 10.1111/apt.15989 32717123

[55] O'Gorman P Monaghan A McGrath M Naimimohasses S Gormley J Norris S . Determinants of physical activity engagement in patients with nonalcoholic fatty liver disease: The need for an individualized approach to lifestyle interventions. Phys Ther (2021) 101(2):pzaa 195. doi: 10.1093/ptj/pzaa195 33104787

[56] Sargeant JA Gray LJ Bodicoat DH Willis SA Stensel DJ Nimmo MA . The effect of exercise training on intrahepatic triglyceride and hepatic insulin sensitivity: a systematic review and meta-analysis. Obes Rev (2018) 19(10):1446–59. doi: 10.1111/obr.12719 30092609

[57] Heyens LJM Busschots D Koek GH Robaeys G Francque S . Liver fibrosis in non-alcoholic fatty liver disease: From liver biopsy to non-invasive biomarkers in diagnosis and treatment. Front Med (2021) 8:615978. doi: 10.3389/fmed.2021.615978 PMC8079659 33937277

[58] Ekstedt M Franzén LE Mathiesen UL Thorelius L Holmqvist M Bodemar G . Long-term follow-up of patients with NAFLD and elevated liver enzymes. Hepatology (2006) 44(4):865–73. doi: 10.1002/hep.21327 17006923

[59] Balkhed W Åberg FO Nasr P Ekstedt M Kechagias S . Repeated measurements of non-invasive fibrosis tests to monitor the progression of non-alcoholic fatty liver disease: A long-term follow-up study. Liver Int (2022) 42(7):1545–56. doi: 10.1111/liv.15255 PMC9314831 35319156

[60] Sabag A Barr L Armour M Armstrong A Baker CJ Twigg SM . The effect of high-intensity interval training vs moderate-intensity continuous training on liver fat: A systematic review and meta-analysis. J Clin Endocrinol Metab (2022) 107(3):862–81. doi: 10.1210/clinem/dgab795 34724062

[61] Bellicha A van Baak MA Battista F Beaulieu K Blundell JE Busetto L . Effect of exercise training on weight loss, body composition changes, and weight maintenance in adults with overweight or obesity: An overview of 12 systematic reviews and 149 studies. Obes Rev (2021) 22(S4):e13256. doi: 10.1111/obr.13256 33955140 PMC8365736

[62] Baker CJ Martinez-Huenchullan SF D'Souza M Xu Y Li M Bi Y . Effect of exercise on hepatic steatosis: Are benefits seen without dietary intervention? a systematic review and meta-analysis. J Diabetes (2021) 13(1):63–77. doi: 10.1111/1753-0407.13086 32667128

[63] Nath P Panigrahi MK Sahu MK Narayan J Sahoo RK Patra AA . Effect of exercise on NAFLD and its risk factors: Comparison of moderate versus low intensity exercise. J Clin Transl Hepatol (2020) 8(2):120–6. doi: 10.14218/JCTH.2019.00012 PMC7438352 32832391

[64] Kerr CJ Waterworth SP Brodie D Sandercock GRH Ingle L . The associations between physical activity intensity, cardiorespiratory fitness, and non-alcoholic fatty liver disease. J Gastroenterol Hepatol (2021) 36(12):3508–14. doi: 10.1111/jgh.15672 34427948

[65] McPherson S Armstrong MJ Cobbold JF Corless L Anstee QM Aspinall RJ . Quality standards for the management of non-alcoholic fatty liver disease (NAFLD): consensus recommendations from the British association for the study of the liver and British society of gastroenterology NAFLD special interest group. Lancet Gastroenterol Hepatol (2022) 7(8):755–69. doi: 10.1016/S2468-1253(22)00061-9 PMC7614852 35490698

[66] Babu AF Csader S Lok J Gómez-Gallego C Hanhineva K El-Nezami H . Positive effects of exercise intervention without weight loss and dietary changes in NAFLD-related clinical parameters: A systematic review and meta-analysis. Nutrients (2021) 13(9):3135. doi: 10.3390/nu13093135 34579012 PMC8466505

[67] Chatterjee A Basu A Das K Singh P Mondal D Bhattacharya B . Hepatic transcriptome signature correlated with HOMA-IR explains early nonalcoholic fatty liver disease pathogenesis. Ann Hepatology (2020) 19(5):472–81. doi: 10.1016/j.aohep.2020.06.009 32682086

[68] Vogeser M König D Frey I Predel H-G Parhofer KG Berg A . Fasting serum insulin and the homeostasis model of insulin resistance (HOMA-IR) in the monitoring of lifestyle interventions in obese persons. Clin Biochem (2007) 40(13):964–8. doi: 10.1016/j.clinbiochem.2007.05.009 17583689

[69] Arab JP Arrese M Trauner M . Recent insights into the pathogenesis of nonalcoholic fatty liver disease. Annu Rev Pathol (2018) 13:321–50. doi: 10.1146/annurev-pathol-020117-043617 29414249

[70] Musso G Gambino R Cassader M . Recent insights into hepatic lipid metabolism in non-alcoholic fatty liver disease (NAFLD). Prog Lipid Res (2009) 48(1):1–26. doi: 10.1016/j.plipres.2008.08.001 18824034

[71] Gordon T Castelli WP Hjortland MC Kannel WB Dawber TR . High density lipoprotein as a protective factor against coronary heart disease. Framingham Study Am J Med (1977) 62(5):707–14. doi: 10.1016/0002-9343(77)90874-9 193398

[72] Bhargava S de la Puente-Secades S Schurgers L Jankowski J . Lipids and lipoproteins in cardiovascular diseases: a classification. Trends Endocrinol Metab (2022) 33(6):409–23. doi: 10.1016/j.tem.2022.02.001 35370062

[73] Austin MA Hokanson JE Edwards KL . Hypertriglyceridemia as a cardiovascular risk factor. Am J Cardiol (1998) 81(4a):7b–12b. doi: 10.1016/s0002-9149(98)00031-9 9526807

[74] Myers J McAuley P Lavie CJ Despres J-P Arena R Kokkinos P . Physical activity and cardiorespiratory fitness as major markers of cardiovascular risk: Their independent and interwoven importance to health status. Prog Cardiovasc Diseases (2015) 57(4):306–14. doi: 10.1016/j.pcad.2014.09.011 25269064

[75] Keating SE Hackett DA Parker HM O'Connor HT Gerofi JA Sainsbury A . Effect of aerobic exercise training dose on liver fat and visceral adiposity. J Hepatol (2015) 63(1):174–82. doi: 10.1016/j.jhep.2015.02.022 25863524

